# Supplementary material for: Gauge fixing for sequence-function relationships
Source: bioRxiv. 2024 Jun 24:2024.05.12.593772. Originally published 2024 May 13. Preprint. [Version 2] doi: 10.1101/2024.05.12.593772 (PMC11118547; doi:10.1101/2024.05.12.593772)
Supplement: Supplement 1 [file media-1.pdf]

Supplemental Information for: Gauge fixing for sequence-function relationships

Anna Posfai, Juannan Zhou, David M. McCandlish, Justin B. Kinney

June 22, 2024

Contents

1 Sequences and embeddings 1

2 Gauge freedoms 1

3 Linear gauges 2

4 All-order interaction models 5

5 Parametric family of gauges 8

6 Trivial gauge, euclidean gauge, and equitable gauge 13

7 Hierarchical gauges 14

8 Hierarchical gauges of an empirical landscape for protein GB1 19

9 Appendix 20

1 Sequences and embeddings

Definition 1. *The alphabet* $\mathcal{A}$ *is an ordered set of* α *characters* $(c_1, \ldots, c_\alpha)$.

Definition 2. *Sequence space* $\mathcal{S}$ *is the set of all sequences of length* L *built from characters in the alphabet* $\mathcal{A}$. *We use* N *to denote the number of sequences in* $\mathcal{S}$, *and* s_l *to denote the character at position* l *in sequence* s.

Definition 3. *An embedding* $\vec{x}$ *is a mapping from* $\mathcal{S}$ *to a real vector space* $V = \mathbb{R}^M$. *We use* M *throughout to denote the dimension of* V.

Definition 4. *The embedding space* S *of an embedding* $\vec{x} : \mathcal{S} \to V$ *is a vector space defined by* $S \equiv \mathrm{span}(\{\vec{x}(s) : s \in \mathcal{S}\})$. S *is also called the span of* $\vec{x}$.

Definition 5. *The design matrix* X *of an embedding* $\vec{x} : \mathcal{S} \to V$ *is an* $N \times M$ *matrix having elements* $X_{ij} = [\vec{x}(s_i)]_j$, *where* $i = 1, \ldots, N$ *indexes all sequences in* $\mathcal{S}$ *(the specific order does not matter) and* $j = 1, \ldots, M$ *indexes the dimensions of* V.

2 Gauge freedoms

Definition 6. *The space of gauge freedoms (a.k.a. freedom space)* G *of an embedding* $\vec{x} : \mathcal{S} \to V$ *is a vector space defined by*

$$G \equiv \left\{ \vec{g} \in V : \vec{g}^\top \vec{x}(s) = 0 \ \forall \ s \in \mathcal{S} \right\}. \tag{1}$$

We use γ *to denote the dimension of* G.

Claim 1. *Let* $\vec{x}$ *be an embedding,* S *be the embedding space of* $\vec{x}$, *and* G *be the freedom space of* $\vec{x}$. *Then* G *is the orthogonal complement of* S, *i.e.,* $G = S^\perp$.

Proof. First we show that $G \subseteq S^{\perp}$. Consider any $\vec{g} \in G$. By Definition 6, $\vec{g}^{\top}\vec{x}(s) = 0$ for every $s \in \mathcal{S}$, and so $\vec{g}$ is orthogonal to S, and thus $\vec{g} \in S^{\perp}$. This establishes that $G \subseteq S^{\perp}$. Next we show that $S^{\perp} \subseteq G$. If $\vec{v} \in S^{\perp}$, then $\vec{v}^{\top}\vec{w} = 0$ for every $\vec{w} \in S$. In particular, $\vec{v}^{\top}\vec{x}(s) = 0$ for every $s \in \mathcal{S}$, implying that $\vec{v} \in G$. This establishes that $S^{\perp} \subseteq G$, proving the claim. □

Gauge freedoms of pairwise-interaction models. Eq. 7 in the main text appears to gives 2α linear relations for every pair of positions $l < l'$, namely

$$x_l^c(s) = \sum_{c' \in \mathcal{A}} x_{ll'}^{cc'}(s) \quad (\alpha \text{ linear relations}), \tag{2}$$

$$x_{l'}^{c'}(s) = \sum_{c \in \mathcal{A}} x_{ll'}^{cc'}(s) \quad (\alpha \text{ linear relations}). \tag{3}$$

However, these linear relations are not independent, as summing Eq. 2 over all $c \in \mathcal{A}$ gives the same linear relation as summing Eq. 3 over all $c' \in \mathcal{A}$:

$$1 = \sum_{c,c' \in \mathcal{A}} x_{ll'}^{cc'}(s). \tag{4}$$

Eq. 4 is in fact the only dependency between the 2α linear relations in Eq. 2 and Eq. 3. Therefore, there are actually $2\alpha - 1$ independent gauge freedoms per pair of positions $l < l'$, and there are $\binom{L}{2}$ such pairs of positions. We thus find a total of

$$\gamma_{\text{pairwise}} = L + \binom{L}{2}(2\alpha - 1) \tag{5}$$

gauge freedoms for the pairwise-interaction model.

3 Linear gauges

Definition 7. *A linear gauge space Θ for an embedding $\vec{x} : \mathcal{S} \to V$ having freedom space G is a vector space such that, for all $\vec{v} \in V$, there is a unique decomposition $\vec{v} = \vec{\theta} + \vec{g}$ where $\vec{\theta} \in \Theta$ and $\vec{g} \in G$. All gauge spaces discussed in what follows are assumed to be linear. Note that $\dim \Theta = M - \gamma$.*

Definition 8. *A projection matrix P that projects from a vector space V into a vector space V_1 along a vector space V_2 is a matrix such that, for all $\vec{v} \in V$, $P\vec{v} \in V_1$ and $\vec{v} - P\vec{v} \in V_2$.*

Claim 2. *Let $\vec{x} : \mathcal{S} \to V$ be an embedding, let G be the freedom space of $\vec{x}$, and let Θ be a gauge space of $\vec{x}$. Then there is a unique a projection matrix P that projects V into Θ along G. Moreover, the matrix $Q \equiv I_M - P$ is the unique matrix that projects V into G along Θ.*

Proof. Let $\vec{e}_1, \ldots, \vec{e}_\gamma$ be a basis for G, and $\vec{f}_1, \ldots, \vec{f}_{M-\gamma}$ be a basis for Θ. Using these basis vectors as columns, define the $M \times \gamma$ matrix $E \equiv (\vec{e}_1, \ldots, \vec{e}_\gamma)$ and the $M \times (M - \gamma)$ matrix $F \equiv (\vec{f}_1, \ldots, \vec{f}_{M-\gamma})$. Choose any $\vec{v} \in V$. By Definition 7, $\vec{v}$ can be uniquely decomposed as

$$\vec{v} = \vec{\theta} + \vec{g} \tag{6}$$

where $\vec{\theta} \in \Theta$ and $\vec{g} \in G$. Because the columns of E and F provide bases for G and Θ, respectively, there is a γ-dimensional vector $\vec{a}$ and an $(M - \gamma)$-dimensional vector $\vec{b}$ such that $\vec{g} = E\vec{a}$ and $\vec{\theta} = F\vec{b}$. Therefore,

$$\vec{v} = E\vec{a} + F\vec{b} \tag{7}$$

$$= (E \quad F)\begin{pmatrix} \vec{a} \\ \vec{b} \end{pmatrix} \tag{8}$$

$$\Rightarrow \begin{pmatrix} \vec{a} \\ \vec{b} \end{pmatrix} = (E \quad F)^{-1}\vec{v} \tag{9}$$

$$\Rightarrow \vec{\theta} = (0_{M\times\gamma} \ F)\begin{pmatrix} \vec{a} \\ \vec{b} \end{pmatrix} \tag{10}$$

$$= (0_{M\times\gamma} \ F)\,(E \quad F)^{-1}\,\vec{v}, \tag{11}$$

where $(E \quad F)$ is the $M \times M$ matrix given by horizontally concatenating E and F, and $0_{M\times\gamma}$ is an $M \times \gamma$ matrix of zeros. A projection matrix P from V into Θ along G therefore exists and is given by

$$P = (0_{M\times\gamma} \ F)\,(E \quad F)^{-1}. \tag{12}$$

To see that Q projects V into G along Θ, simply note that, for any $\vec{v} \in V$, $Q\vec{v} = \vec{v} - P\vec{v} \in G$ and $\vec{v} - Q\vec{v} = P\vec{v} \in \Theta$. To prove that P is unique, assume that there is another matrix $P' \neq P$ that projects into Θ along G. There must therefore be a $\vec{v} \in V$ such that $P'\vec{v} \neq P\vec{v}$. By Definition 8, $P'\vec{v} \in \Theta$ and $\vec{v} - P'\vec{v} \in G$. But $P\vec{v} \in \Theta$ and $\vec{v} - P\vec{v} \in G$ as well, and by Definition 7, the decomposition of $\vec{v}$ into a component in Θ and a component in G is unique, implying that $P\vec{v} = P'\vec{v}$, which is a contradiction. An analogous proof shows that Q is unique. □

Definition 9. *A metric Λ on an M-dimensional vector space V is a symmetric positive-definite $M \times M$ matrix. The Λ-inner product of two vectors $\vec{v}, \vec{w} \in V$ is defined to be*

$$\langle \vec{v}, \vec{w} \rangle_\Lambda \equiv \vec{v}^\top \Lambda \vec{w}. \tag{13}$$

The Λ-norm of a vector $\vec{v} \in V$ is defined to be

$$||\vec{v}||_\Lambda \equiv \sqrt{\langle \vec{v}, \vec{v} \rangle_\Lambda} = \sqrt{\vec{v}^\top \Lambda \vec{w}}. \tag{14}$$

$\vec{v}$ and $\vec{w}$ are Λ-orthogonal if and only if $\langle \vec{v}, \vec{w} \rangle_\Lambda = 0$.

Definition 10. *An orthogonalizing metric, Λ, for a gauge space Θ and freedom space G is a metric for which all $\vec{g} \in G$ are Λ-orthogonal to all $\vec{\theta} \in \Theta$.*

Claim 3. *Let $\vec{x} : \mathcal{S} \to V$ be an embedding, G be the freedom space of $\vec{x}$, and Θ be a gauge space of $\vec{x}$. Then there exists an orthogonalizing metric Λ for Θ and G.*

Proof. Let P be the projection matrix from V into Θ along G, let $Q = I_M - P$, and define the matrix $\Lambda \equiv P^\top P + Q^\top Q$. It is a simple matter to show that Λ is symmetric and positive-definite, and is thus a metric. Using $P^2 = P$, $Q^2 = Q$ and $Q = I_M - P$, it is readily shown that PQ are both equal to the zero matrix and hence Λ satisfies $\Lambda = P^\top \Lambda P + Q^\top \Lambda Q$. Therefore, by Claim 25 in the Appendix, Λ orthogonalizes Θ and G. □

Claim 4. *Let $\vec{x} : \mathcal{S} \to V$ be an embedding, X be the design matrix of $\vec{x}$, G be the freedom space of $\vec{x}$, Θ be a gauge space of $\vec{x}$, and Λ be a metric that orthogonalizes Θ and G. Then for any vector $\vec{\theta}_{\text{init}} \in V$, the vector $\vec{\theta}^*$ in the gauge orbit of $\vec{\theta}_{\text{init}}$ that has minimal Λ-norm lies in Θ.*

Proof. Let θ^* be the unique element of Θ that lies in the gauge orbit of $\vec{\theta}_{\text{init}}$. Because Λ orthogonalizes Θ and G, $\vec{g}^\top \Lambda \vec{\theta}^* = 0$ for all $\vec{g} \in G$. By Claim 25,

$$||\vec{\theta}^* + \vec{g}||_\Lambda^2 \geq ||\vec{\theta}^*||_\Lambda^2, \tag{15}$$

with equality obtaining only when $\vec{g} = \vec{0}$. This proves that $\vec{\theta}^*$ is the unique vector with the smallest Λ-norm of any vector in the gauge orbit of $\vec{\theta}_{\text{init}}$. □

Claim 5. *Let $\vec{x} : \mathcal{S} \to V$ be an embedding, X be the design matrix of $\vec{x}$, G be the freedom space of $\vec{x}$, Θ be a gauge space of $\vec{x}$, and Λ be a metric that orthogonalizes Θ and G. Then the matrix P that projects along G into Θ is given by $P = \Lambda^{-1/2}(X\Lambda^{-1/2})^+ X$.*

Proof. Consider the transformed embedding $\vec{x}' = \Lambda^{-1/2}\vec{x}$, which corresponds to the transformed design matrix $X' = X\Lambda^{-1/2}$ as well as the transformed embedding space $S' = \Lambda^{-1/2}S$. If a parameter vector $\vec{\theta} \in V$ yields model predictions $X\vec{\theta}$), then $\vec{\theta}' = \Lambda^{1/2}\vec{\theta}$ yields the same model predictions $X'\vec{\theta}'$. Consequently, $G' = \Lambda^{1/2}G$ is the transformed freedom space and $\Theta' = \Lambda^{-1/2}\Theta$ is the transformed gauge space. Because Θ and G are Λ-orthogonal, Θ' and G' are orthogonal in the Euclidean sense. The transformed embedding space S' is also orthogonal to G', and so $\Theta' = S'$ The matrix P' that projects along G' and onto Θ' is therefore the orthogonal projection matrix onto the space spanned by the rows of X', and is given by $P' = (X')^+ X$. Now consider an initial parameter $\vec{\theta}_{\text{init}} \in V$, its gauge-fixed counterpart $\vec{\theta}_{\text{fixed}} = P\vec{\theta}_{\text{init}} \in \Theta$ as well as the transformed versions of these vectors, $\vec{\theta}'_{\text{init}} = \Lambda^{-1/2}\vec{\theta}_{\text{init}}$ and $\vec{\theta}'_{\text{fixed}} = \Lambda^{-1/2}\vec{\theta}_{\text{fixed}} \in \Theta'$. One can readily verify that

$$X\vec{\theta}_{\text{init}} = X\vec{\theta}_{\text{fixed}} = X'\vec{\theta}'_{\text{init}} = X'\vec{\theta}'_{\text{fixed}}. \tag{16}$$

Therefore,

$$\begin{aligned}
\vec{\theta}_{\text{fixed}} &= \Lambda^{-1/2}\vec{\theta}'_{\text{fixed}}, && (17)\\
&= \Lambda^{-1/2}P'\vec{\theta}'_{\text{init}}, && (18)\\
&= \Lambda^{-1/2}(X')^+ X'\vec{\theta}'_{\text{init}}, && (19)\\
&= \Lambda^{-1/2}(X\Lambda^{-1/2})^+ X\Lambda^{-1/2}\Lambda^{1/2}\vec{\theta}_{\text{init}}, && (20)\\
&= P\vec{\theta}_{\text{init}} && (21)
\end{aligned}$$

where $P = \Lambda^{-1/2}(X\Lambda^{-1/2})^+ X$. This proves the claim. □

Definition 11. *A loss function $\mathcal{L}$ is said to be L_2-regularized by a metric Λ iff it has the form*

$$\mathcal{L}(\vec{\theta}) = \mathcal{L}_{\text{data}}(X\vec{\theta}) + \frac{\beta}{2}\vec{\theta}^\top \Lambda \vec{\theta}, \tag{22}$$

where X is the design matrix of an embedding $\vec{x}$, $\mathcal{L}_{\text{data}}$ is a data-dependent loss function that depends only on model predictions $X\vec{\theta}$, and β is a positive scalar.

Claim 6. *Let $\vec{x} : \mathcal{S} \to V$ be an embedding, G be the freedom space of $\vec{x}$, Θ be a gauge space of $\vec{x}$, Λ be a metric that orthogonalizes Θ and G, $\mathcal{L}$ be a loss function that is L_2-regularized by Λ, and $\vec{\theta}^*$ be a minimum of $\mathcal{L}$. Then $\vec{\theta}^* \in \Theta$.*

Proof.

$$\begin{aligned}
\vec{\theta}^* &= \operatorname{argmin}_{\vec{v}\in V}\ \mathcal{L}(\vec{v}) && (23)\\
&= \operatorname{argmin}_{\vec{v}\in V}\ \left[\mathcal{L}_{\text{data}}(X\vec{v}) + \frac{\beta}{2}\vec{v}^\top\Lambda\vec{v}\right] && (24)\\
&= \operatorname{argmin}_{\left\{\vec{v}=\vec{\theta}+\vec{g}:\vec{\theta}\in\Theta,\,\vec{g}\in G\right\}}\ \left[\mathcal{L}_{\text{data}}(X(\vec{\theta}+\vec{g})) + \frac{\beta}{2}(\vec{\theta}+\vec{g})^\top\Lambda(\vec{\theta}+\vec{g})\right] && (25)\\
&= \operatorname{argmin}_{\vec{\theta}\in\Theta}\ \left[\mathcal{L}_{\text{data}}(X\vec{\theta}) + \frac{\beta}{2}\vec{\theta}^\top\Lambda\vec{\theta}\right] + \operatorname{argmin}_{\vec{g}\in G}\ \left[\frac{\beta}{2}\vec{g}^\top\Lambda\vec{g}\right] && (26)\\
&= \operatorname{argmin}_{\vec{\theta}\in\Theta}\ \left[\mathcal{L}_{\text{data}}(X\vec{\theta}) + \frac{\beta}{2}\vec{\theta}^\top\Lambda\vec{\theta}\right]. && (27)
\end{aligned}$$

In Eq. 25 we used the fact that $\vec{v} \in V$ can be expressed uniquely as $\vec{v} = \vec{\theta} + \vec{g}$ for $\vec{\theta} \in \Theta$ and $\vec{g} \in G$ (by Definition 7), and in Eq. 26 we used the fact that $X\vec{g} = \vec{0}$ for any $\vec{g} \in G$ (by Definition 6), together with the assumption that Λ orthogonalizes Θ and G. We thus find that $\vec{\theta}^* \in \Theta$. □

Claim 7. *Let $\vec{x} : \mathcal{S} \to V$ be an embedding, G be the freedom space of $\vec{x}$, Θ be a gauge space of $\vec{x}$, $\mathcal{L}_{\text{data}}$ be a data-dependent loss function that depends only on model predictions $X\vec{\theta}$, and Δ be an $N \times N$ positive definite matrix. Then there exists a metric Λ defining a loss $\mathcal{L}$ that is L_2-regularized by Λ that has the following property: every minimum $\vec{\theta}^*$ of $\mathcal{L}$ lies within Θ and satisfies*

$$\mathcal{L}(\theta^*) = \mathcal{L}_{\text{data}}(X\vec{\theta}^*) + \frac{\beta}{2}\left(X\vec{\theta}^*\right)^\top \Delta \left(X\vec{\theta}^*\right). \tag{28}$$

Proof. By Claim 6 it suffices to construct a Λ that is an orthogonalizing metric for Θ and G and which satisfies Equation 28. Let P be the projection matrix from V into Θ along G, let $Q = I_M - P$, and define the matrix $\Lambda \equiv P^\top X^\top \Delta X P + Q^\top Q$. It is a simple matter to show that Λ is symmetric and positive-definite, and is thus a metric. Using $P^2 = P$ and $Q^2 = Q$, and $Q = I_M - P$, it is readily shown that PQ are both equal to the zero matrix and hence Λ satisfies $\Lambda = P^\top \Lambda P + Q^\top \Lambda Q$. Therefore, by Claim 25 in the Appendix, Λ orthogonalizes Θ and G. Then by Claim 6, we have $\vec{\theta}^* \in \Theta$ and hence $P\vec{\theta}^* = \vec{\theta}^*$ and $\vec{\theta}^*$ is in the null space of Q. Consequently,

$$\begin{aligned}
\mathcal{L}(\theta^*) &= \mathcal{L}_{\text{data}}(X\vec{\theta}^*) + \frac{\beta}{2}\vec{\theta}^{*\top}\left(P^\top X^\top \Delta X P + Q^\top Q\right)\vec{\theta}^* && (29)\\
&= \mathcal{L}_{\text{data}}(X\vec{\theta}^*) + \frac{\beta}{2}\left(\vec{\theta}^{*\top} P^\top X^\top \Delta X P \vec{\theta}^* + \vec{\theta}^{*\top} Q^\top Q \vec{\theta}^*\right) && (30)\\
&= \mathcal{L}_{\text{data}}(X\vec{\theta}^*) + \frac{\beta}{2}\left(X\vec{\theta}^*\right)^\top \Delta \left(X\vec{\theta}^*\right) && (31)
\end{aligned}$$

as required. □

Practical recipe for fixing a linear gauge using L_2 regularization. Claim 7 shows that for any choice of linear gauge Θ and any desired positive definite L_2 regularizer Δ on model predictions, we can construct a positive definite L_2 regularizer Λ on model parameters that penalizes the vector of predictions according to Δ and results in an inferred parameter vector $\vec{\theta}^*$ that is guaranteed to be a member of our desired gauge Θ. Practically, the steps to calculate Λ are:

1. Find a basis $\vec{e}_1, \ldots, \vec{e}_\gamma$ for the null space of the design matrix X. This can be done, for instance, by using Gaussian elimination to column reduce the block matrix $\begin{pmatrix} X \\ I_M \end{pmatrix}$, where the resulting matrix will have γ non-zero columns whose top N entries are 0 and whose bottom N entries will each serve as one vector in the desired basis. See see ref. [1] for analytical methods to determine this basis for the all-order interaction model and related models.

2. Find a basis $\vec{f}_1, \ldots, \vec{f}_{M-\gamma}$ for the desired linear gauge space Θ. Any linearly independent set of $M - \gamma$ vectors that are members of Θ will suffice.

3. Using these basis vectors as columns, define the $M \times \gamma$ matrix $E \equiv (\vec{e}_1, \ldots, \vec{e}_\gamma)$ and the $M \times (M-\gamma)$ matrix $F \equiv (\vec{f}_1, \ldots, \vec{f}_{M-\gamma})$. Then calculate the projection matricies $P = (0_{M\times\gamma} \; F)\,(E \;\; F)^{-1}$ and $Q = I_M - P$.
4. Set $\Lambda \equiv P^\top X^\top \Delta X P + Q^\top Q$.
5. Minimize $\mathcal{L}(\vec{\theta}) \equiv \mathcal{L}_{\text{data}}(X\vec{\theta}) + \frac{\beta}{2}\vec{\theta}^\top \Lambda \vec{\theta}$ for some choice of regularization parameter $\beta > 0$.

Note similarly that given a specified L_2 regularizer Λ on model parameters, the induced L_2 regularizer on model predictions is given by $\Delta \equiv (PX^+)^\top \Lambda (PX^+)$ which satisfies $x^\top \Delta x > 0$ for all nonzero x in the column space of X.

4 All-order interaction models

Definition 12. *The position-specific augmented embedding $\vec{x}'_l : \mathcal{S} \to V_l$, where $V_l = \mathbb{R}^{\alpha+1}$, is given by*

$$\vec{x}'_l(s) = \begin{pmatrix} x_l^*(s) \\ x_l^{c_1}(s) \\ \cdots \\ x_l^{c_\alpha}(s) \end{pmatrix} \quad \text{for all} \;\; s \in \mathcal{S}, \tag{32}$$

where, as in the main text, $x_l^*(s) = 1$ for all $s \in \mathcal{S}$ and, for all $c \in \mathcal{A}$, $x_l^c(s)$ is one if $s_l = c$ and is zero otherwise. In what follows, we use G_l to denote the freedom space of $\vec{x}'_l$ and S_l to denote the embedding space of $\vec{x}'_l$.

Claim 8. *S_l has dimension α and is given by $\left\{(\beta^*, \beta^{c_1}, \ldots, \beta^{c_\alpha})^\top : \beta^* = \sum_{c\in\mathcal{A}} \beta^c\right\}$; G_l has dimension 1 and is spanned by the vector $(-1, 1, \ldots, 1)^\top$.*

Proof. Let S_l denote the span of $\vec{x}'_l$. By definition, any vector $\vec{v} \in S_l$ can be written as a linear combination of vectors $\vec{x}'_l(s)$ over $s \in S$, i.e.,

$$\vec{v} = \sum_{s\in\mathcal{S}} \beta(s) \begin{pmatrix} x_l^*(s) \\ x_l^{c_1}(s) \\ \vdots \\ x_l^{c_\alpha}(s) \end{pmatrix} \tag{33}$$

for some mapping $\beta : \mathcal{S} \to \mathbb{R}$. Defining $\beta^c \equiv \sum_{s\in\mathcal{S}} \beta(s) x_l^c(s)$ for all $c \in \mathcal{A}'$, we get

$$\vec{v} = \begin{pmatrix} \beta^* \\ \beta^{c_1} \\ \vdots \\ \beta^{c_\alpha} \end{pmatrix}. \tag{34}$$

The $\alpha + 1$ values $\{\beta^c\}_{c\in\mathcal{A}}$ are arbitrary except for one constraint arising from the fact that $x_l^*(s) = \sum_{c\in\mathcal{A}} x_l^c(s)$ for all $s \in \mathcal{S}$:

$$\begin{aligned} \sum_{c\in\mathcal{A}} \beta^c &= \sum_{c\in\mathcal{A}} \sum_{s\in\mathcal{S}} \beta(s) x_l^c(s) && (35) \\ &= \sum_{s\in\mathcal{S}} \beta(s) \sum_{c\in\mathcal{A}} x_l^c(s) && (36) \\ &= \sum_{s\in\mathcal{S}} \beta(s) x_l^*(s) && (37) \\ &= \beta^*. && (38) \end{aligned}$$

We therefore see that S_l has dimension α and comprises the vectors stated in Claim 8.

Now let $\vec{g} = (-1, 1, \ldots, 1)^\top$. Taking the dot product of $\vec{g}$ with $\vec{x}'_l$ gives

$$\vec{g}^\top \vec{x}'(s) = -x_l^*(s) + \sum_{c\in\mathcal{A}} x_l^c(s) = 0 \;\; \text{for all} \;\; s \in \mathcal{S}, \tag{39}$$

This shows that $\vec{g} \in G_l$ and, in light of the fact that $\dim G_l = \dim V - \dim S_l = 1$, proves that G_l is spanned by $\vec{g}$. □

Definition 13. *The all-order embedding $\vec{x}_{\text{all}} : \mathcal{S} \to V_{\text{all}}$ ($V_{\text{all}} = \mathbb{R}^M$ where $M = (\alpha+1)^L$) is defined by the tensor product*

$$\vec{x}_{\text{all}}(s) = \bigotimes_{l=1}^{L} \vec{x}'_l(s) \quad \text{for all} \;\; s \in \mathcal{S}. \tag{40}$$

We use S_{all} to denote the embedding space of $\vec{x}_{\text{all}}$, and G_{all} to denote the freedom space of $\vec{x}_{\text{all}}$.

Claim 9. *The embedding space of $\vec{x}_{\text{all}}$ is given by*

$$S_{\text{all}} = \bigotimes_{l=1}^{L} S_l. \tag{41}$$

Proof. For each $c \in \mathcal{A}$, define α distinct $(\alpha+1)$-dimensional vectors $\vec{e}_c$, one for each $c \in \mathcal{A}$ and with elements indexed by $c' \in \mathcal{A}'$ given by

$$[\vec{e}_c]_{c'} = \begin{cases} 1 & \text{if } c' = * \text{ or } c' = c \\ 0 & \text{otherwise} \end{cases} \tag{42}$$

Note that, for any $s \in \mathcal{S}$, $\vec{e}_{s_l} = \vec{x}'_l(s)$. Next, for each position l, define the set of vectors $\mathcal{B}_l = \{\vec{e}_c\}_{c\in\mathcal{A}}$. From the proof of Claim 8, $\mathcal{B}_l$ forms a linearly independent basis for S_l. By definition of the tensor product of vector spaces, a basis for the space $S \equiv \bigotimes_{l=1}^{L} S_l$ is given by

$$\begin{aligned}
\mathcal{B} &\equiv \left\{ \bigotimes_{l=1}^{L} \vec{v}_l : \vec{v}_1 \in \mathcal{B}_1, \ldots, \vec{v}_L \in \mathcal{B}_L \right\} && (43)\\
&= \left\{ \bigotimes_{l=1}^{L} \vec{e}_{c_l} : c_1, \ldots, c_L \in \mathcal{A} \right\} && (44)\\
&= \left\{ \bigotimes_{l=1}^{L} \vec{e}_{s_l} : s \in \mathcal{S} \right\} && (45)\\
&= \left\{ \bigotimes_{l=1}^{L} \vec{x}'_l(s) : s \in \mathcal{S} \right\} && (46)\\
&= \{\vec{x}_{\text{all}}(s) : s \in \mathcal{S}\}. && (47)
\end{aligned}$$

$\mathcal{B}$ is therefore also a basis for S_{all}. Since S and S_{all} share the same basis, they are the same vector space. Note that we have learned, in the process, that all vectors $\vec{x}_{\text{all}}(s)$, $s \in \mathcal{S}$, are linearly independent. □

Claim 10. *The freedom space of $\vec{x}_{\text{all}}$ is given by*

$$G_{\text{all}} = \bigoplus_{(R_1,\ldots,R_L)\in\mathcal{R}} \left[\bigotimes_{l=1}^{L} R_l \right], \tag{48}$$

where

$$\mathcal{R} = \{(R_1, \ldots, R_L) : R_l \in \{S_l, G_l\} \text{ for all } l \text{ and } R_l = G_l \text{ for at least one } l\}. \tag{49}$$

Proof.

$$\begin{aligned}
V_{\text{all}} &= \bigotimes_{l=1}^{L} V_l && (50)\\
&= \bigotimes_{l=1}^{L} [S_l \oplus G_l] && (51)\\
&= \left[\bigotimes_{l=1}^{L} S_l \right] \oplus \left[\bigoplus_{\{R_1,\ldots,R_L\}\in\mathcal{R}} \bigotimes_{l=1}^{L} R_l \right] && (52)\\
&= S_{\text{all}} \oplus W && (53)
\end{aligned}$$

where

$$W \equiv \bigoplus_{\{R_1,\ldots,R_L\}\in\mathcal{R}} \bigotimes_{l=1}^{L} R_l, \tag{54}$$

This shows that $\dim W = \dim V_{\text{all}} - \dim S_{\text{all}} = \dim G_{\text{all}}$. However, this does not yet show that $W = G_{\text{all}}$.

To see that $W = G_{\text{all}}$, let $\{\vec{u}_l^c\}_{c\in\mathcal{A}'}$ be a basis for V_l. Each $\vec{u}_l^c$ has a unique decomposition $\vec{u}_l^c = \vec{\theta}_l^c + \vec{g}_l^c$ where $\vec{\theta}_l^c \in S_l$ and $\vec{g}_l^c \in G_l$. For every augmented sequence $s' \in \mathcal{S}'$, there is a corresponding vector in V_{all} given by $\vec{u}_{s'} = \bigotimes_{l=1}^{L} \vec{u}_l^{s_l'}$. Moreover,

the set $\{\vec{u}_{s'}\}_{s'\in\mathcal{S}'}$ is a basis for V_{all}. The vector $\vec{u}_{s'}$ can be decomposed into a component in S_{all} and a component in W via

$$\begin{aligned}
\vec{u}_{s'} &= \bigotimes_{l=1}^{L} \vec{u}_l^{s'_l} && (55)\\
&= \bigotimes_{l=1}^{L} \left[\vec{\theta}_l^{s'_l} + \vec{g}_l^{s'_l}\right] && (56)\\
&= \bigotimes_{l=1}^{L} \vec{\theta}_l^{s'_l} + \sum_{(\vec{r}_1,\ldots,\vec{r}_L)\in\rho_{s'}} \bigotimes_{l=1}^{L} \vec{r}_l && (57)\\
&= \vec{\theta}_{s'} + \vec{r}_{s'} && (58)
\end{aligned}$$

where

$$\begin{aligned}
\vec{\theta}_{s'} &\equiv \bigotimes_{l=1}^{L} \vec{\theta}_l^{s'_l}, && (59)\\
\vec{r}_{s'} &\equiv \sum_{(\vec{r}_1,\ldots,\vec{r}_L)\in\rho_{s'}} \bigotimes_{l=1}^{L} \vec{r}_l, && (60)\\
\rho_{s'} &\equiv \left\{(\vec{r}_1,\ldots,\vec{r}_L) : \vec{r}_l \in \left\{\vec{\theta}_l^{s'_l}, \vec{g}_l^{s'_l}\right\} \text{ for all } l, \text{ and } \vec{r}_l = \vec{g}_l^{s'_l} \text{ for at least one } l\right\}. && (61)
\end{aligned}$$

Observe that $r_{s'} \in W$ for every $s' \in \mathcal{S}'$. Moreover, since $\{\vec{u}_{s'}\}_{s'\in\mathcal{S}'}$ is a basis for $\vec{V}_{\text{all}}$, $\{\vec{r}_{s'}\}_{s'\in\mathcal{S}'}$ is a basis for W. Finally, observe that for every $s \in \mathcal{S}$, the dot product of $\vec{x}_{\text{all}}(s)$ with $\vec{r}_{s'}$ is zero, i.e.,

$$[\vec{x}_{\text{all}}(s)]^\top \vec{r}_{s'} = \sum_{(\vec{r}_1,\ldots,\vec{r}_L)\in\rho_{s'}} \prod_{l=1}^{L} [\vec{x}'_l(s)]^\top \vec{r}_l = 0, \tag{62}$$

because $\vec{r}_l \in G_l$ for at least one l in each product. Therefore, $\vec{r}_{s'} \in G_{\text{all}}$ for every $s' \in \mathcal{S}'$. Since $\{\vec{r}_{s'}\}_{s'\in\mathcal{S}'}$ is a basis for W, we conclude that $W = G_{\text{all}}$.

□

Claim 11. *For each position l, let Θ_l be a gauge space for $\vec{x}'_l$, let P_l be projection matrix into Θ_l along G_l, and let Λ_l be a metric that orthogonalizes Θ_l and G_l. Then $\Theta \equiv \bigotimes_l \Theta_l$ is a gauge space of $\vec{x}_{\text{all}}$, $P \equiv \bigotimes_l P_l$ projects into Θ along G_{all}, and $\Lambda \equiv \bigotimes_l \Lambda_l$ is a metric that orthogonalizes Θ and G_{all}.*

Proof. For each position $l = 1,\ldots,L$, let $\vec{v}_l = \vec{\theta}_l + \vec{g}_l$ where $\vec{v}_l \in V_l$, $\vec{\theta}_l \in \Theta_l$, and $\vec{g}_l \in G_l$. Defining $\vec{v} = \bigotimes_{l=1}^{L} \vec{v}_l$, we get

$$\vec{v} = \bigotimes_{l=1}^{L} \left[\vec{\theta}_l + \vec{g}_l\right] = \vec{\theta} + \vec{g} \tag{63}$$

where $\vec{\theta} = \bigotimes_{l=1}^{L} \vec{\theta}_l$ lives in the space $\Theta \equiv \bigotimes_{l=1}^{L} \Theta_l$, and

$$\begin{aligned}
\vec{g} &= \sum_{(\vec{r}_1,\ldots,\vec{r}_L)\in\rho} \bigotimes_{l=1}^{L} \vec{r}_l, && (64)\\
\rho &\equiv \left\{(\vec{r}_1,\ldots,\vec{r}_L) : \vec{r}_l \in \left\{\vec{\theta}_l, \vec{g}_l\right\} \text{ for all } l, \text{ and } \vec{r}_l = \vec{g}_l \text{ for at least one } l\right\}, && (65)
\end{aligned}$$

lives in the space

$$G \equiv \bigoplus_{(R_1,\ldots,R_L)\in\mathcal{R}} \left[\bigotimes_{l=1}^{L} R_l\right], \tag{66}$$

where

$$\mathcal{R} = \{(R_1,\ldots,R_L) : R_l \in \{\Theta_l, G_l\} \text{ for all } l \text{ and } R_l = G_l \text{ for at least one } l\}. \tag{67}$$

By arguments similar to those in Claim 10, it is readily seen that $\vec{x}_{\text{all}}(s)^\top \vec{g} = \vec{0}$ for every $s \in \mathcal{S}$. Moreover, by arguments similar to those in Claim 10, it is readily seen that this construction is able to yield a basis of vectors $\vec{g}$ for G, proving

that that $G = G_{\text{all}}$ is the freedom space of $\vec{x}_{\text{all}}$. Therefore, for every vector of the form $\vec{v} = \bigotimes_{l=1}^{L} \vec{v}_l$, there is a unique decomposition $\vec{v} = \vec{\theta} + \vec{g}$, where $\vec{\theta} \in \Theta$ and $\vec{g} \in G_{\text{all}}$. Because a basis of such vectors $\vec{v}$ can be found for V, we see that any vector $\vec{v} \in V$ can be uniquely decomposed as $\vec{v} = \vec{\theta} + \vec{g}$, where $\vec{\theta} \in \Theta$ and $\vec{g} = G_{\text{all}}$. This proves that Θ is a gauge space of $\vec{x}_{\text{all}}$.

Defining $P \equiv \bigotimes_{l=1}^{L} P_l$ and applying this to $\vec{v}$, we find that

$$P\vec{v} = \bigotimes_{l=1}^{L} P_l \vec{v}_l = \bigotimes_{l=1}^{L} \vec{\theta}_l = \vec{\theta} \tag{68}$$

is in Θ, which implies that $\vec{v} - P\vec{v} = \vec{g}$ is in G_{all}. Since a basis of vectors $\vec{v}$ for V can be found that decompose in this way, all vectors in V decompose in this manner. This proves that P projects into Θ along G_{all}

Now define $\Lambda \equiv \bigotimes_{l=1}^{L} \Lambda_l$. It is readily seen that Λ is symmetric and positive definite from the fact that every Λ_l is symmetric and positive definite. Moreover,

$$\begin{aligned}
\Lambda &= \bigotimes_{l=1}^{L} (P_l^\top \Lambda_l P_l + Q_l^\top \Lambda_l Q_l) & (69)\\
&= \bigotimes_{l=1}^{L} P_l^\top \Lambda_l P_l + \sum_{(R_1,\ldots,R_L)\in\mathcal{R}} \bigotimes_{l=1}^{L} R_l^\top \Lambda_l R_l & (70)\\
&= \left[\bigotimes_{l=1}^{L} P_l\right]^\top \left[\bigotimes_{l=1}^{L} \Lambda_l\right] \left[\bigotimes_{l=1}^{L} P_l\right] + \sum_{(R_1,\ldots,R_L)\in\mathcal{R}} \left[\bigotimes_{l=1}^{L} R_l\right]^\top \left[\bigotimes_{l=1}^{L} \Lambda_l\right] \left[\bigotimes_{l=1}^{L} R_l\right] & (71)\\
&= \left[\bigotimes_{l=1}^{L} P_l\right]^\top \left[\bigotimes_{l=1}^{L} \Lambda_l\right] \left[\bigotimes_{l=1}^{L} P_l\right] + \left[\sum_{(R_1,\ldots,R_L)\in\mathcal{R}} \bigotimes_{l=1}^{L} R_l\right]^\top \left[\bigotimes_{l=1}^{L} \Lambda_l\right] \left[\sum_{(R_1,\ldots,R_L)\in\mathcal{R}} \bigotimes_{l=1}^{L} R_l\right] & (72)\\
&= P^\top \Lambda P + Q^\top \Lambda Q. & (73)
\end{aligned}$$

where $Q = I_M - P$ and the set $\mathcal{R}$ of ordered operator sets is defined to be

$$\mathcal{R} \equiv \{(R_1, \ldots, R_L) : R_i = P_i \text{ or } R_i = Q_i \text{ for all } i = 1, \ldots, L, \text{ with } R_i = Q_i \text{ for at least one } i\}. \tag{74}$$

In Eq. 69, we used the fact that Λ_l satisfies

$$\Lambda_l = P_l^\top \Lambda_l P_l^\top + Q_l^\top \Lambda_l Q_l, \tag{75}$$

where $Q_l \equiv I_{(\alpha+1)} - P_l$ (see Claim 25). In going from Eq. 71 to Eq. 72, we used the fact that, if two ordered sets of operators $(R_1, \ldots, R_L)$ and $(R'_1, \ldots, R'_L)$ in $\mathcal{R}$ are different, then

$$\left[\bigotimes_{l=1}^{L} R_l\right]^\top \left[\bigotimes_{l=1}^{L} \Lambda_l\right] \left[\bigotimes_{l=1}^{L} R'_l\right] = \bigotimes_{l=1}^{L} R_l^\top \Lambda R'_l = 0 \tag{76}$$

since there will be an l such that $R_l^\top \Lambda_l R'_l$ is either $P_l^\top \Lambda_l Q_l = 0$ or $Q_l \Lambda_l P_l = 0$. In going from Eq. 72 to Eq. 73, we used the fact that

$$\sum_{(R_1,\ldots,R_L)\in\mathcal{R}} \bigotimes_{l=1}^{L} R_l = \bigotimes_{i=1}^{L} (P_l + Q_l) - \bigotimes_{i=1}^{L} P_l = I_M - P = Q. \tag{77}$$

The fact that P projects into Θ along G_{all}, together with the result $\Lambda = P^\top \Lambda P + Q^\top \Lambda Q$ in Eq. 73 proves (by Claim 25) that Λ orthogonalizes Θ and G_{all}.

□

5 Parametric family of gauges

Definition 14. *A probability distribution p on $\mathcal{S}$ is positive iff $p(s) > 0$ for all $s \in \mathcal{S}$.*

Definition 15. *A factorizable probability distribution p on $\mathcal{S}$ is one that can be written $p(s) = \prod_{l=1}^{L} p_l^{s_l}$ for all $s \in \mathcal{S}$, where p_l is a probability distribution over the α possible characters at position l, i.e., $p(s) = p_l^{s_l}$.*

Definition 16. *An augmented sequence s' is a sequence built from the augmented alphabet $\mathcal{A}' = \{*, c_1, \ldots, c_\alpha\}$, where $c_1, \ldots, c_\alpha$ are the characters in $\mathcal{A}$ and $*$ is a wild-card character that is interpreted as matching any character in $\mathcal{A}$. The set of all augmented sequences is denoted $\mathcal{S}'$. Note that every augmented sequence s' is can be interpreted as a subset of $\mathcal{S}$ that comprises sequences matching the pattern defined by s'. For this reason we will sometimes use expressions like $s \in s'$ and $s' \subseteq t'$ (for $s \in \mathcal{S}$ and $s', t' \in \mathcal{S}$).*

To aid in our discussion of the all-order interaction model [Eq. **??**], we define an augmented alphabet $\mathcal{A}' = \{*, c_1, \ldots, c_\alpha\}$, where $c_1, \ldots, c_\alpha$ are the characters in $\mathcal{A}$ and $*$ is a wild-card character that is interpreted as matching any character in $\mathcal{A}$. Let $\mathcal{S}'$ denote the set of sequences of length L comprising characters from $\mathcal{A}'$. For each augmented sequence $s' \in \mathcal{S}'$, we define the sequence feature $x_{s'}(s)$ to be 1 if a sequence s matches the pattern described by s' and to be 0 otherwise. In this way, each augmented sequence s' serves as a regular expression against which bona fide sequences are compared.

Definition 17. *Given a non-negative real number λ, a factorizable probability distribution p on $\mathcal{S}$, and a sequence position l, the position-specific parametric gauge $\Theta_l^{\lambda,p}$ is defined as*

$$\Theta_l^{\lambda,p} \equiv V_\lambda \oplus V_\perp^{p_l}, \tag{78}$$

where $V_\lambda \equiv \operatorname{span}\{(\lambda, 1, \cdots, 1)^\top\}$ and $V_\perp^{p_l} \equiv \left\{(0, v_{c_1}, \ldots, v_{c_\alpha})^\top : \sum_{c\in\mathcal{A}} p_l^c v_c = 0\right\}$.

Claim 12. *The matrix $P_l^{\lambda,p}$ that projects V_l along G_l and onto $\Theta_l^{\lambda,p}$ is an $(\alpha+1)\times(\alpha+1)$ matrix given by*

$$P_l^{\lambda,p} = \begin{pmatrix} \eta & p_l^{c_1}\eta & \cdots & p_l^{c_\alpha}\eta \\ 1-\eta & 1-p_l^{c_1}\eta & \cdots & -p_l^{c_\alpha}\eta \\ \vdots & \vdots & \ddots & \vdots \\ 1-\eta & -p_l^{c_1}\eta & \cdots & 1-p_l^{c_\alpha}\eta \end{pmatrix}, \tag{79}$$

where $\eta \equiv \lambda/(1+\lambda)$.

Proof. Any $(\alpha+1)$-dimensional vector $\vec{v} \in V_l$ can be decomposed as

$$\vec{v} = \vec{\theta} + \vec{g}, \tag{80}$$

where $\vec{\theta} \in \Theta_l^{\lambda,p}$ and $\vec{g} \in G_l$. From the definitions of $\Theta_l^{\lambda,p}$ and G, $\vec{\theta}$ and $\vec{g}$ must have the forms

$$\vec{\theta} = c_\lambda \vec{v}_\lambda + \vec{\theta}_\perp \quad \text{and} \quad \vec{g} = c_{-1}\vec{e}_{-1}, \tag{81}$$

where

$$\vec{e}_\lambda = \begin{pmatrix} \lambda \\ 1 \\ \vdots \\ 1 \end{pmatrix}, \quad \vec{e}_{-1} = \begin{pmatrix} -1 \\ 1 \\ \vdots \\ 1 \end{pmatrix}, \quad \vec{\theta}_\perp = \begin{pmatrix} 0 \\ \theta_l^{c_1} \\ \vdots \\ \theta_l^{c_\alpha} \end{pmatrix}, \tag{82}$$

and where c_λ and c_{-1} are real numbers that depend on $\vec{v}$. The projection matrix $Q_l^{\lambda,p} \equiv I - P_l^{\lambda,p}$ projects V_l into G_l along $\Theta_l^{\lambda,p}$, and so is related to the scalar c_{-1} via

$$Q_l^{\lambda,p}\vec{v} = c_{-1}\vec{e}_{-1}. \tag{83}$$

We now compute c_{-1} as a function of $\vec{v}$. First we define the metric

$$\Lambda \equiv \begin{pmatrix} 1 & 0 & \cdots & 0 \\ 0 & \alpha p_l^{c_1} & \cdots & 0 \\ \vdots & \vdots & \ddots & \vdots \\ 0 & 0 & \cdots & \alpha p_l^{c_\alpha} \end{pmatrix}, \tag{84}$$

and compute

$$\vec{e}_\lambda^\top \Lambda \vec{e}_\lambda = \lambda^2 + \sum_{i=1}^{\alpha} \alpha p_l^{c_i} = \alpha + \lambda^2, \tag{85}$$

$$\vec{e}_{-1}^\top \Lambda \vec{e}_\lambda = -\lambda + \sum_{i=1}^{\alpha} \alpha p_l^{c_i} = \alpha - \lambda, \tag{86}$$

$$\vec{e}_{-1}^{\top}\Lambda\vec{e}_{-1} = 1+\sum_{i=1}^{\alpha}\alpha p_l^{c_i} = \alpha+1, \tag{87}$$

$$\vec{e}_{\lambda}^{\top}\Lambda\vec{\theta}_{\perp} = 0+\sum_{i=1}^{\alpha}\alpha p_l^{c_i}\theta_l^{c_i} = 0, \tag{88}$$

$$\vec{e}_{-1}^{\top}\Lambda\vec{\theta}_{\perp} = 0+\sum_{c}\sum_{i=1}^{\alpha}\alpha p_l^{c_i}\theta_l^{c_i} = 0. \tag{89}$$

Next we solve for c_{-1} via

$$\begin{aligned}
\vec{e}_{\lambda}^{\top}\Lambda\vec{v} &= c_{\lambda}(\vec{e}_{\lambda}^{\top}\Lambda\vec{e}_{\lambda}) + c_{-1}(\vec{e}_{\lambda}^{\top}\Lambda\vec{e}_{-1}) + (\vec{e}_{\lambda}^{\top}\Lambda\vec{\theta}_{\perp}) &(90)\\
&= c_{\lambda}(\alpha+\lambda^2) + c_{-1}(\alpha-\lambda). &(91)\\
\vec{e}_{-1}^{\top}\Lambda\vec{v} &= c_{\lambda}(\vec{e}_{-1}^{\top}\Lambda\vec{e}_{\lambda}) + c_{-1}(\vec{e}_{-1}^{\top}\Lambda\vec{e}_{-1}) + (\vec{e}_{-1}^{\top}\Lambda\vec{\theta}_{\perp}) &(92)\\
&= c_{\lambda}(\alpha-\lambda) + c_{-1}(\alpha+1). &(93)\\
\Rightarrow (\alpha-\lambda)\vec{e}_{\lambda}^{\top}\Lambda\vec{v} - (\alpha+\lambda^2)\vec{e}_{-1}^{\top}\Lambda\vec{v} &= c_{-1}\left[(\alpha-\lambda)^2 - (\alpha+1)(\alpha+\lambda^2)\right] &(94)\\
&= c_{-1}\left[-2\alpha\lambda - \alpha(1+\lambda^2)\right] &(95)\\
&= -\alpha(1+2\lambda+\lambda^2)c_{-1} &(96)\\
&= -\alpha(1+\lambda)^1 c_{-1} &(97)\\
\Rightarrow c_{-1} &= -\frac{(\alpha-\lambda)}{\alpha(1+\lambda)^2}\vec{e}_{\lambda}^{\top}\Lambda\vec{v} + \frac{(\alpha+\lambda^2)}{\alpha(1+\lambda)^2}\vec{e}_{-1}^{\top}\Lambda\vec{v}. &(98)
\end{aligned}$$

This shows that $Q_l^{\lambda,p}$ is given by

$$Q_l^{\lambda,p} = -\frac{(\alpha-\lambda)}{\alpha(1+\lambda)^2}\vec{e}_{-1}\vec{e}_{\lambda}^{\top}\Lambda + \frac{(\alpha+\lambda^2)}{\alpha(1+\lambda)^2}\vec{e}_{-1}\vec{e}_{-1}^{\top}\Lambda. \tag{99}$$

Next we compute the matrix elements $[Q_l^{\lambda,p}]_{ij}$ for all $i,j\in\{0,1,\ldots,\alpha\}$. Setting $i=0$, $j=0$ gives

$$\begin{aligned}
[Q_l^{\lambda,p}]_{00} &= -\frac{(\alpha-\lambda)}{\alpha(1+\lambda)^2}(-\lambda) + \frac{(\alpha+\lambda^2)}{\alpha(1+\lambda)^2}(1) &(100)\\
&= \frac{(\alpha-\lambda)\lambda + (\alpha+\lambda^2)}{\alpha(1+\lambda)^2} &(101)\\
&= \frac{\alpha(1+\lambda)}{\alpha(1+\lambda)^2} &(102)\\
&= \frac{1}{1+\lambda} &(103)\\
&= 1-\eta. &(104)
\end{aligned}$$

Setting $i=0$, $j>0$ gives

$$\begin{aligned}
[Q_l^{\lambda,p}]_{0j} &= -\frac{(\alpha-\lambda)}{\alpha(1+\lambda)^2}(-\alpha p_l^{c_j}) + \frac{(\alpha+\lambda^2)}{\alpha(1+\lambda)^2}(-\alpha p_l^{c_j}) &(105)\\
&= \frac{\alpha(\alpha-\lambda) - \alpha(\alpha+\lambda^2)}{\alpha(1+\lambda)^2}p_l^{c_j} &(106)\\
&= -\frac{\lambda(1+\lambda)}{(1+\lambda)^2}p_l^{c_j} &(107)\\
&= -\frac{\lambda}{1+\lambda}p_l^{c_j} &(108)\\
&= -\eta p_l^{c_j}. &(109)
\end{aligned}$$

Setting $i>0$, $j=0$ gives

$$\begin{aligned}
[Q_l^{\lambda,p}]_{i0} &= -\frac{(\alpha-\lambda)}{\alpha(1+\lambda)^2}(\lambda) + \frac{(\alpha+\lambda^2)}{\alpha(1+\lambda)^2}(-1) &(110)\\
&= \frac{-\lambda(\alpha-\lambda) - (\alpha+\lambda^2)}{\alpha(1+\lambda)^2} &(111)
\end{aligned}$$

$$= -\frac{\alpha(1+\lambda)}{\alpha(1+\lambda)^2} \quad (112)$$

$$= -\frac{1}{1+\lambda} \quad (113)$$

$$= \eta - 1. \quad (114)$$

Setting $i > 0$, $j > 0$ gives

$$[Q_l^{\lambda,p}]_{ij} = -\frac{(\alpha-\lambda)}{\alpha(1+\lambda)^2}(\alpha p_l^{c_j}) + \frac{(\alpha+\lambda^2)}{\alpha(1+\lambda)^2}(\alpha p_l^{c_j}) \quad (115)$$

$$= \frac{-(\alpha-\lambda)+(\alpha+\lambda^2)}{(1+\lambda)^2} p_l^{c_j} \quad (116)$$

$$= \frac{\lambda(1+\lambda)}{(1+\lambda)^2} p_l^{c_j} \quad (117)$$

$$= \frac{\lambda}{1+\lambda} p_l^{c_j} \quad (118)$$

$$= \eta p_l^{c_j}. \quad (119)$$

We therefore obtain

$$Q_l^{\lambda,p} = \begin{pmatrix} 1-\eta & -p_l^{c_1}\eta & \cdots & -p_l^{c_\alpha}\eta \\ \eta-1 & p_l^{c_1}\eta & \cdots & p_l^{c_\alpha}\eta \\ \vdots & \vdots & \ddots & \vdots \\ \eta-1 & p_l^{c_1}\eta & \cdots & p_l^{c_\alpha}\eta \end{pmatrix}. \quad (120)$$

Finally, plugging this result into $P_l^{\lambda,p} = I - Q_l^{\lambda,p}$ gives

$$P_l^{\lambda,p} = \begin{pmatrix} \eta & p_l^{c_1}\eta & \cdots & p_l^{c_\alpha}\eta \\ 1-\eta & 1-p_l^{c_1}\eta & \cdots & -p_l^{c_\alpha}\eta \\ \vdots & \vdots & \ddots & \vdots \\ 1-\eta & -p_l^{c_1}\eta & \cdots & 1-p_l^{c_\alpha}\eta \end{pmatrix}, \quad (121)$$

which proves the claim. □

Claim 13. *$\Theta^{\lambda,p} \equiv \bigotimes_l \Theta_l^{\lambda,p}$ is a valid gauge space for the embedding $\vec{x}_{\text{all}}$.*

Proof. This follows from Claim 11 and the fact that $\Theta_l^{\lambda,p}$ is a valid gauge space for $\vec{x}_l$. □

Claim 14. *The matrix $P^{\lambda,p}$ that projects V along G_{all} and into $\Theta^{\lambda,p}$ is an $M \times M$ matrix with elements*

$$P_{s't'}^{\lambda,p} = \prod_{\substack{l \text{ s.t.} \\ s'_l \in \mathcal{A} \\ t'_l \in \mathcal{A}}} \left(\delta_{s'_l t'_l} - p_l^{t'_l}\eta\right) \times \prod_{\substack{l \text{ s.t.} \\ s'_l = * \\ t'_l \in \mathcal{A}}} \left(p_l^{t'_l}\eta\right) \times \prod_{\substack{l \text{ s.t.} \\ s'_l \in \mathcal{A} \\ t'_l = *}} (1-\eta) \times \prod_{\substack{l \text{ s.t.} \\ s'_l = * \\ t'_l = *}} \eta. \quad (122)$$

Proof. By Claim 11, $\Theta^{\lambda,p} \equiv \bigotimes_l \Theta_l^{\lambda,p}$ imples that $P^{\lambda,p} \equiv \bigotimes_l P_l^{\lambda,p}$. Eq. 122 follows from expressing the elements of $P_l^{\lambda,p}$ in Eq. 79 as

$$[P^{\lambda,p}]_{cc'} = \begin{cases} \delta_{cc'} - p_l^{c'}\eta & \text{if } c \in \mathcal{A}, c' \in \mathcal{A}, \\ p_l^{c'}\eta & \text{if } c = *, c' \in \mathcal{A}, \\ 1-\eta & \text{if } c \in \mathcal{A}, c' = *, \\ \eta & \text{if } c = *, c' = *, \end{cases} \quad (123)$$

then taking the product across positions l using $c = s'_l$ and $c' = t'_l$. □

Claim 15. *If λ and p are positive, the $\alpha \times \alpha$ metric*

$$\Lambda_l^{\lambda,p} \equiv \begin{pmatrix} 1 & 0 & \cdots & 0 \\ 0 & \lambda p_l^{c_1} & \cdots & 0 \\ \vdots & \vdots & \ddots & \vdots \\ 0 & 0 & \cdots & \lambda p_l^{c_\alpha} \end{pmatrix} \quad (124)$$

orthogonalizes $\Theta_l^{\lambda,p}$ and G_l.

Proof. Assume that $\Lambda_l^{\lambda,p}$ is diagonal, and thus has the form

$$\Lambda_l^{\lambda,p} = \begin{pmatrix} d_0 & 0 & \cdots & 0 \\ 0 & d_1 & \cdots & 0 \\ \vdots & \vdots & \ddots & \vdots \\ 0 & 0 & \cdots & d_\alpha \end{pmatrix}, \tag{125}$$

for some set of scalars $d_0, \ldots, d_\alpha$. The requirement that $\Lambda_l^{\lambda,p}$ be a metric (and thus positive definite) implies that all $d_0, \ldots, d_\alpha$ must be positive. We now solve for $d_0, \ldots, d_\alpha$ using the matrix equation (Claim 25):

$$\Lambda_l^{\lambda,p} = \left(P_l^{\lambda,p}\right)^\top \Lambda_l^{\lambda,p} \left(P_l^{\lambda,p}\right) + \left(Q_l^{\lambda,p}\right)^\top \Lambda_l^{\lambda,p} \left(Q_l^{\lambda,p}\right), \tag{126}$$

$$\Rightarrow d_i\delta_{ij} = \sum_{k=0}^{\alpha} d_k [P_l^{\lambda,p}]_{ki} [P_l^{\lambda,p}]_{kj} + \sum_{k=0}^{\alpha} d_k [Q_l^{\lambda,p}]_{ki} [Q_l^{\lambda,p}]_{kj}, \tag{127}$$

where $Q_l^{\lambda,p} \equiv I - P_l^{\lambda,p}$. We now solve Eq. 127 for different choices of i and j using the matrix elements of $P_l^{\lambda,p}$ and $Q_l^{\lambda,p}$ computed in the proof of Claim 12. For $i = j = 0$, we get

$$\begin{aligned}
d_0 &= \left[d_0[P_l^{\lambda,p}]_{00}^2 + \sum_{k=1}^{\alpha} d_k [P_l^{\lambda,p}]_{k0}^2\right] + \left[d_0[Q_l^{\lambda,p}]_{00}^2 + \sum_{k=1}^{\alpha} d_k [Q_l^{\lambda,p}]_{k0}^2\right] &(128)\\
&= \left[d_0\eta^2 + \sum_{k=1}^{\alpha} d_k (1-\eta)^2\right] + \left[d_0(1-\eta)^2 + \sum_{k=1}^{\alpha} d_k(\eta-1)^2\right] &(129)\\
&= [d_0\eta^2 + a(1-\eta)^2] + [d_0(1-\eta)^2 + a(\eta-1)^2] &(130)\\
&= d_0[2\eta^2 - 2\eta + 1] + 2a(1-\eta)^2, &(131)\\
\Rightarrow 2a(2-\eta)^2 &= 2d_0\eta(1-\eta), &(132)\\
\Rightarrow a &= d_0\frac{\eta}{1-\eta} &(133)\\
&= d_0\lambda, &(134)
\end{aligned}$$

where in Eq. 134 we have used $\lambda = \eta/(1-\eta)$. For $i = 0$, $j > 0$, we get

$$\begin{aligned}
0 &= \left[d_0[P_l^{\lambda,p}]_{00}[P_l^{\lambda,p}]_{0j} + \sum_{k=1}^{\alpha} d_k [P_l^{\lambda,p}]_{k0}[P_l^{\lambda,p}]_{kj}\right] + \left[d_0[Q_l^{\lambda,p}]_{00}[Q_l^{\lambda,p}]_{0j} + \sum_{k=1}^{\alpha} d_k [Q_l^{\lambda,p}]_{k0}[Q_l^{\lambda,p}]_{kj}\right] &(135)\\
&= \left[d_0p_j\eta^2 + \sum_{k=1}^{\alpha} d_k(1-\eta)(\delta_{jk} - p_j\eta)\right] + \left[-d_0p_j\eta(1-\eta) + \sum_{k=1}^{\alpha} d_k(\eta-1)(p_j\eta)\right] &(136)\\
&= 2d_0p_j\eta^2 - d_0p_j\eta - 2ap_j\eta(1-\eta) + d_j(1-\eta) &(137)\\
&= 2(d_0+a)p_j\eta^2 - (2a+d_0)p_j\eta + d_j(1-\eta) &(138)\\
&= 2d_0(1+\lambda)\frac{\lambda}{1+\lambda}p_j(\eta-1) + d_0p_j\eta + d_j(1-\eta), &(139)\\
\Rightarrow d_j &= 2d_0p_j\lambda + d_0p_j\frac{\eta}{\eta-1} &(140)\\
&= 2d_0p_j\lambda - d_0p_j\lambda &(141)\\
&= d_0p_j\lambda. &(142)
\end{aligned}$$

The resulting equation,

$$d_i = d_0p_i\lambda, \quad \text{for all} \quad i = 1, \ldots, \alpha, \tag{143}$$

determines all elements of $\Lambda_l^{\lambda,p}$ up to a multiplicative factor d_0. However, we must still verify that Eq. 143 is consistent with the constraints placed on the other elements of $\Lambda_l^{\lambda,p}$ by Eq. 127. For $i > 0$, $j = 0$, we get the same result as for $i = 0$, $j > 0$, because Eq. 127 is symmetric. For $i > 0$, $j > 0$, $i = j$, we get

$$\begin{aligned}
d_i &= \left[d_0[P_l^{\lambda,p}]_{0i}^2 + \sum_{k=1}^{\alpha} d_k [P_l^{\lambda,p}]_{ki}^2\right] + \left[d_0[Q_l^{\lambda,p}]_{0i}^2 + \sum_{k=1}^{\alpha} d_k [Q_l^{\lambda,p}]_{ki}^2\right] &(144)\\
&= \left[d_0p_i^2\eta^2 + \sum_{k=1}^{\alpha} d_k(\delta_{ki} - p_i\eta)^2\right] + \left[d_0p_i^2\eta^2 + \sum_{k=1}^{\alpha} d_kp_i^2\eta^2\right] &(145)
\end{aligned}$$

$$
\begin{aligned}
&= \left[d_0 p_i^2\eta^2 + d_i - 2d_i p_i\eta + a p_i^2\eta^2\right] + \left[d_0 p_i^2\eta^2 + a p_i^2\eta^2\right] && (146)\\
&= d_i + 2[(d_0+a)p_i^2\eta^2 - d_i p_i\eta], && (147)\\
\Rightarrow d_i &= (d_0+a)p_i\eta && (148)\\
&= d_0(1+\lambda)\frac{\lambda}{1+\lambda}p_i && (149)\\
&= d_0 p_i\lambda, && (150)
\end{aligned}
$$

which is consistent with Eq. 143. For $i>0$, $j>0$, $i\neq j$, we get

$$
\begin{aligned}
0 &= \left[d_0[P_l^{\lambda,p}]_{0i}[P_l^{\lambda,p}]_{0j} + \sum_{k=1}^{\alpha} d_k[P_l^{\lambda,p}]_{ki}[P_l^{\lambda,p}]_{kj}\right]\\
&\quad + \left[d_0[Q_l^{\lambda,p}]_{0i}[Q_l^{\lambda,p}]_{0j} + \sum_{k=1}^{\alpha} d_k[Q_l^{\lambda,p}]_{ki}[Q_l^{\lambda,p}]_{kj}\right] && (151)\\
&= \left[d_0 p_i p_j\eta^2 + \sum_{k=1}^{\alpha} d_k(\delta_{ki}-p_i\eta)(\delta_{kj}-p_j\eta)\right] + \left[d_0 p_i p_j + \sum_{k=1}^{\alpha} d_k p_i p_j\eta^2\right] && (152)\\
&= 2d_0 p_i p_j\eta^2 + 2a p_i p_j\eta^2 - d_i p_j\eta - p_i d_j\eta && (153)\\
&= 2(d_0+a)p_i p_j\eta^2 - d_i p_j\eta - p_i d_j\eta, && (154)\\
\Rightarrow d_i p_j + p_i d_j &= 2d_0(1+\lambda)\frac{\lambda}{1+\lambda} && (155)\\
&= 2d_0\lambda, && (156)
\end{aligned}
$$

which is also consistent with Eq. 143. We therefore see that Eq. 143 is indeed consistent with Eq. 127. Thus we find that

$$
\Lambda_l^{\lambda,p} = d_0\begin{pmatrix} 1 & 0 & \cdots & 0\\ 0 & \lambda p_1 & \cdots & 0\\ \vdots & \vdots & \ddots & \vdots\\ 0 & 0 & \cdots & \lambda p_\alpha \end{pmatrix}, \qquad (157)
$$

orthogonalizes $\Theta_l^{\lambda,p}$ and G_l, and is determined up to an unknown positive scalar d_0. This proves the claim. □

Claim 16. *If λ and p are positive, the $M\times M$ metric*

$$
\Lambda_{s't'} \equiv p(s')\lambda^{o(s')}\delta_{s't'} \qquad (158)
$$

orthogonalizes $\Theta^{\lambda,p}$ and G_{all}.

Proof. By Claim 11, $\Theta^{\lambda,p}\equiv\bigotimes_l\Theta_l^{\lambda,p}$ implies that $\Lambda^{\lambda,p}\equiv\bigotimes_l\Lambda_l^{\lambda,p}$. Eq. 158 follows from expressing the elements of $\Lambda_l^{\lambda,p}$ in Eq. 157 as

$$
[\Lambda_l^{\lambda,p}]_{cc'} = \delta_{cc'}p_l^c \times \begin{cases} 1 & \text{if } c=*,\\ \lambda & \text{if } c\in\mathcal{A}, \end{cases} \qquad (159)
$$

then taking the product across positions l using $c=s'_l$ and $c'=t'_l$. □

6 Trivial gauge, euclidean gauge, and equitable gauge

Claim 17. *The trivial gauge $\Theta^{0,p}$ is unaffected by the probability distribution p.*

Proof. Setting $\lambda=0$ gives $\eta=0$, and thus

$$
\begin{aligned}
P_{s't'}^{0,p} &= \prod_{\{l:s'_l\in\mathcal{A}\text{ and }t'_l\in\mathcal{A}\}}\delta_{s'_lt'_l} \times \prod_{\{l:s'_l=*\}} 0 && (160)\\
&= \prod_{\{l:s'_l\in\mathcal{A}\text{ and }t'_l\in\mathcal{A}\}}\delta_{s'_lt'_l} \times \begin{cases} 1 & \text{if } s'\in\mathcal{S}\\ 0 & \text{otherwise}\end{cases} && (161)\\
&= \prod_{\{l:t'_l\in\mathcal{A}\}}\delta_{s'_lt'_l} \times \begin{cases} 1 & \text{if } s'\in\mathcal{S}\\ 0 & \text{otherwise}\end{cases} && (162)
\end{aligned}
$$

$$\Rightarrow P^{0,p}_{s't'} = \begin{cases} 1 & \text{if } s' \in \mathcal{S} \text{ and } x_{t'}(s') = 1 \\ 0 & \text{otherwise} \end{cases} , \tag{163}$$

which does not depend on p. □

Claim 18. *The euclidean gauge is equal to the embedding space, i.e.,* $\Theta_{\text{eucl}} = S_{\text{all}}$. *Moreover standard* L_2 *regularization yields parameters in* Θ_{eucl}.

Proof. In the Euclidean gauge $p(s) = \alpha^{-L}$ for all $s \in \mathcal{S}$. Consequently, $p(s') = \alpha^{-o(s')}$ for all $s' \in \mathcal{S}'$. Because $\lambda = \alpha$ in this gauge as well, the metric Λ is given by

$$\Lambda_{s't'} = \delta_{s't'} p(s') \lambda^{o(s')} = \delta_{s't'} \alpha^{-o(s')} \alpha^{o(s')} = \delta_{s't'}. \tag{164}$$

Λ is therefore the euclidean metric. Because Θ_{eucl} is Λ-orthogonal to G_{all},

$$\Theta_{\text{eucl}} = G^{\perp}_{\text{all}} = S_{\text{all}}. \tag{165}$$

And because standard L_2 parameter regularization uses a penalty of $\sum_{s'} \theta^2_{s'} = ||\vec{\theta}||^2$, which is the euclidean norm of $\vec{\theta}$, inference using standard L_2 regularization yields parameters in the euclidean gauge. This completes the proof. □

Claim 19. *In the equitable gauge,* $||\vec{\theta}||^2_{\Lambda} = \sum_{s'} p(s')\theta^2_{s'} = \sum_{s'} \left\langle f^2_{s'} \right\rangle_p$ *where* $f_{s'} \equiv \theta_{s'} x_{s'}$.

Proof. The equitable gauge is defined by $\lambda = 1$. Setting $\lambda = 1$ gives $\Lambda_{s't'} = \delta_{s't'} p(s')$, and so

$$||\theta||^2_{\Lambda} = \sum_{s',t'} \Lambda_{s't'} \theta_{s'} \theta_{t'} \tag{166}$$

$$= \sum_{s'} p(s') \theta^2_{s'} \tag{167}$$

$$= \sum_{s'} \langle x_{s'} \rangle_p \theta^2_{s'} \tag{168}$$

$$= \sum_{s'} \left\langle x^2_{s'} \right\rangle_p \theta^2_{s'} \tag{169}$$

$$= \sum_{s'} \left\langle (\theta_{s'} x_{s'})^2 \right\rangle_p \tag{170}$$

$$= \sum_{s'} \left\langle f^2_{s'} \right\rangle_p \tag{171}$$

This completes the proof. □

7 Hierarchical gauges

Definition 18. *The order* $o(s')$ *of an augmented sequence* $s' \in \mathcal{S}'$ *is defined to be the number of non-star characters in* s', *i.e., the number of positions* l *for which* $s_l \in \mathcal{A}$.

Definition 19. *For any two augmented sequences* $s', t' \in \mathcal{S}'$, *we write* $t' \subseteq s'$ *if the sequences matched by* t' *form a subset of those matched by* s'. *More formally,* $t' \subseteq s'$ *iff, for all positions* l, $s'_l \in \mathcal{A} \Rightarrow t'_l = s'_l$. *Note that* $t' \subseteq s'$ *implies that* $o(t') \geq o(s')$.

Definition 20. *For any two augmented sequences* $s', t' \in \mathcal{S}'$, *we write* $t' \succeq s'$ *iff, for all positions* l, $s'_l \in \mathcal{A} \Rightarrow t'_l \in \mathcal{A}$, *or equivalently,* $t'_l = * \Rightarrow s'_l = *$. *Note that* $t' \succeq s'$ *implies that* $o(t') \geq o(s')$. *Also note that* $t' \subseteq s' \Rightarrow t' \succeq s'$, *but that the reverse is not true, since* $t' \succeq s'$ *does not require that* $t'_l = s'_l$ *when* $s'_l \in \mathcal{A}$.

For example, if $L = 8$ and $\mathcal{A} = \{\text{A}, \text{C}, \text{G}, \text{T}\}$ is the set of DNA bases, then

$$**\text{AT}**\text{C}* \succeq ***\text{T}**\text{C}*, \tag{172}$$

$$**\text{AT}**\text{C}* \subseteq ***\text{T}**\text{C}*, \tag{173}$$

whereas,

$$**\text{AT}**\text{G}* \succeq ***\text{T}**\text{C}*, \tag{174}$$

$$**\text{AT}**\text{G}* \not\subseteq ***\text{T}**\text{C}*. \tag{175}$$

Definition 21. *A hierarchical model is a an all-order interaction model in which* $\theta_{s'} = 0$ *for all augmented sequences* s' *in a set* $\mathcal{Z}' \subseteq \mathcal{S}'$ *(the "zero set" of the model) that has the following property: for every* $s' \in \mathcal{Z}'$ *and* $t' \in \mathcal{S}'$*,* $t' \succeq s'$ *implies that* $t' \in \mathcal{Z}'$*.*

Claim 20. *Hierarchical gauges preserve the form of hierarchical models. Specifically, if* $\vec{\theta}$ *are the parameters of a hierarchical model having zero set* $\mathcal{Z}'$*, then* $\vec{\theta}_{\text{fixed}} = P^{\infty,p}\vec{\theta}$ *are also parameters of a hierarchical model with zero set* $\mathcal{Z}'$*.*

Proof. Setting $\lambda = \infty$ in Eq. 22 of the main text, we see that the elements of $P^{\infty,p}_{s't'}$ can written

$$P^{\infty,p}_{s't'} = \prod_{\substack{l \text{ s.t.} \\ s'_l \in \mathcal{A} \\ t'_l \in \mathcal{A}}} \left(\delta_{s'_l t'_l} - p_l^{t'_l}\right) \times \prod_{\substack{l \text{ s.t.} \\ s'_l = * \\ t'_l \in \mathcal{A}}} p_l^{t'_l} \times \prod_{\substack{l \text{ s.t.} \\ s'_l \in \mathcal{A} \\ t'_l = *}} 0, \tag{176}$$

$$= \prod_{\substack{l \text{ s.t.} \\ s'_l \in \mathcal{A} \\ t'_l \in \mathcal{A}}} \left(\delta_{s'_l t'_l} - p_l^{t'_l}\right) \times \prod_{\substack{l \text{ s.t.} \\ s'_l = * \\ t'_l \in \mathcal{A}}} p_l^{t'_l} \times \begin{cases} 1 & \text{if } t' \succeq s' \\ 0 & \text{otherwise} \end{cases} \tag{177}$$

$$= \prod_{\{l : s'_l \in \mathcal{A}\}} \left(\delta_{s'_l t'_l} - p_l^{t'_l}\right) \times \prod_{\{l : s'_l = *\}} p_l^{t'_l} \times \begin{cases} 1 & \text{if } t' \succeq s' \\ 0 & \text{otherwise} \end{cases} . \tag{178}$$

Here we used Def. 20 in going from Eq. 176 to Eq. 177, and we used the fact that $t' \succeq s'$ implies that $s'_l \in \mathcal{A} \Rightarrow t'_l \in \mathcal{A}$, as well as the fact that $t'_l = * \Rightarrow p_l^{t'_l} = 1$, in going from Eq. 177 to Eq. 178. Now assume $\vec{\theta}$ are the parameters of a hierarchical model with zero set $\mathcal{Z}'$, and choose any $s' \in \mathcal{Z}'$. Then in the hierarchical gauge $\Theta^{\infty,p}_{s't'}$,

$$\theta^{\text{fixed}}_{s'} = \sum_{t' \in \mathcal{S}'} P^{\infty,p}_{s't'} \theta_{t'}. \tag{179}$$

$$= \sum_{t' \succeq s'} \theta_{t'} \prod_{\{l : s'_l \in \mathcal{A}\}} \left(\delta_{s'_l t'_l} - p_l^{t'_l}\right) \prod_{\{l : s'_l = *\}} p_l^{t'_l} \tag{180}$$

$$= 0 \tag{181}$$

because $t' \succeq s'$ implies that $t' \in \mathcal{Z}'$ and thus that $\theta_{t'} = 0$ for all t' in the sum. We conclude that $\theta^{\text{fixed}}_{s'} = 0$ for every $s' \in \mathcal{Z}'$, i.e., $\vec{\theta}_{\text{fixed}}$ are the parameters of a hierarchical model defined by zero set $\mathcal{Z}'$. □

Claim 21. *Parameters* $\vec{\theta}_{\text{fixed}}$ *in the hierarchical gauge* $\Theta^{\infty,p}$ *satisfy the marginalization constraint*

$$\sum_{c_k} p_{l_k}^{c_k} \theta^{c_1 \ldots c_K}_{l_1 \ldots l_K, \text{fixed}} = 0 \tag{182}$$

for every $K = 1, \ldots, L$*, every subset of positions* $\{l_1, \ldots, l_K\}$*, and every choice of* $k = 1, \ldots, K$*.*

Proof. From Eq. 180 in the proof of Claim 20, parameters $\vec{\theta}$ in the hierarchical gauge $\Theta^{\infty,p}$ are given in terms of unfixed parameters $\vec{\theta}$ via

$$\theta^{\text{fixed}}_{s'} = \sum_{t' \in \mathcal{S}'} P^{\infty,p}_{s't'} \theta_{t'} = \sum_{t' \succeq s'} \theta_{t'} \prod_{\{l : s'_l \in \mathcal{A}\}} \left(\delta_{s'_l t'_l} - p_l^{t'_l}\right) \prod_{\{l : s'_l = *\}} p_l^{t'_l}. \tag{183}$$

Now choose any $K \in \{1, \ldots, L\}$, any set of positions $\sigma = \{l_1, \ldots, l_K\}$, and any index $k \in \{1, \ldots, K\}$. Define $u' \in \mathcal{S}'$ to be the augmented sequence for which $u'_{l_i} = c_i$ for all $i = 1, \ldots, K$, and that has $u'_l = *$ for all $l \notin \sigma$. Further define $\mathcal{S}_{u',k} \subseteq \mathcal{S}'$ to bet the set of augmented sequences obtained by replacing the character at position k in u' with the α different characters in $\mathcal{A}$. To reduce the notational burden, we use i as a synonym for l_i when $i = 1, \ldots, K$, and use $i = K + 1, \ldots, L$ to denote positions not in σ. We find that

$$\sum_{c_k} p_k^{c_k} \theta^{c_1 \ldots c_K}_{1 \ldots K, \text{fixed}} = \sum_{s' \in \mathcal{S}_{u',k}} p_k^{s'_k} \theta_{s'} \tag{184}$$

$$= \sum_{s' \in \mathcal{S}_{u',k}} p_k^{s'_k} \sum_{t' \subseteq s'} \theta_{t'} \prod_{i=1}^{K} \left(\delta_{s'_i t'_i} - p_i^{t'_i}\right) \prod_{i=K+1}^{L} p_i^{t'_i} \tag{185}$$

$$= \sum_{t' \subseteq u'} \sum_{s' \in \mathcal{S}_{u',k}} \theta_{t'} p_k^{s'_k} \prod_{i=1}^{K} \left(\delta_{s'_i t'_i} - p_i^{t'_i}\right) \prod_{i=K+1}^{L} p_i^{t'_i} \tag{186}$$

$$= \sum_{t'\subseteq u'} \sum_{c\in\mathcal{A}} \theta_{t'} p_k^c (\delta_{ct'_k} - p_k^{t'_k}) \prod_{\substack{i=1\\ i\neq k}}^{K} \Big(\delta_{u'_i t'_i} - p_i^{t'_i}\Big) \prod_{i=K+1}^{L} p_i^{t'_i} \tag{187}$$

$$= \sum_{t'\subseteq u'} \theta_{t'} \left[\sum_{c\in\mathcal{A}} p_k^c (\delta_{ct'_k} - p_k^{t'_k})\right] \prod_{\substack{i=1\\ i\neq k}}^{K} \Big(\delta_{u'_i t'_i} - p_i^{t'_i}\Big) \prod_{i=K+1}^{L} p_i^{t'_i} \tag{188}$$

$$= 0. \tag{189}$$

In going from Eq. 184 to Eq. 185 we used Eq. 183. In going from Eq. 185 to Eq. 186 we used the fact that $t' \subseteq s'$ and $t' \subseteq u'$ are the same condition on t' when $s' \in \mathcal{S}_{u',k}$. In going from Eq. 186 to Eq. 187, we eliminated s' by separating the case $i = k$ out of the product over $i = 1, \ldots, K$, by replacing $s'_k \to c$, and by replacing $s'_i \to u'_i$ for all $i \neq k$. In going from Eq. 187 to Eq. 188, we collected in brackets all quantities that depend on c. And in going from Eq. 188 to Eq. 189, we use the fact that the term in brackets vanishes:

$$\sum_{c\in\mathcal{A}} p_k^c \left(\delta_{ct'_k} - p_k^{t'_k}\right) = \left(\sum_{c\in\mathcal{A}} p_k^c \delta_{ct'_k}\right) - \left(\sum_{c\in\mathcal{A}} p_k^c\right) p_k^{t'_k} = p_k^{t'_k} - p_k^{t'_k} = 0. \tag{190}$$

This proves the claim. □

Definition 22. *The $\mathcal{A}$-positions of an augmented sequence s' are the positions l such that $s'_l \in \mathcal{A}$. Similarly, the $*$-positions of s' are the positions l such that $s'_l = *$.*

Definition 23. *An augmented sequence orbit σ is a set comprising all augmented sequences that have a specified set of $\mathcal{A}$-positions (or equivalently, a specified set of $*$-positions). The order of the orbit, $o(\sigma)$, is defined to be the order of all $s' \in \sigma$. The term "orbit" comes from the fact that such sets are formed from the orbit of s' under the group of position-specific character permutations; see ref. [1].*

Claim 22. *Let $f(s) = \sum_{t'} \theta_{t'} x_{t'}(s)$ be an activity landscape and p be a positive probability distribution. Define the expectation value of f with respect to p conditioned on $s' \in \mathcal{S}'$ to be $\langle f|s'\rangle_p = \frac{1}{p(s')}\sum_{s\in s'} p(s) f(s)$. Then when $\vec{\theta}$ is in the hierarchical gauge,*

$$\langle f|s'\rangle_p = \sum_{t'\supseteq s'} \theta_{t'}. \tag{191}$$

This claim is readily extended to non-positive probability distributions p by defining $\langle f|s'\rangle_p = \lim_{\epsilon\to 0^+} \langle f|s'\rangle_{p_\epsilon}$, where p_ϵ is a regularized version of p given by

$$p_\epsilon(s) = \prod_l \left[(1-\epsilon)p_l^{s_l} + \frac{\epsilon}{\alpha}\right], \tag{192}$$

with p_l being the position-specific factors of p.

Proof. Assume that p is positive, and that $\vec{\theta}$ is in the hierarchical gauge. Then,

$$\langle f|s'\rangle_p = \frac{1}{p(s')} \sum_{s\in s'} p(s) \sum_{t'} \theta_{t'} x_{t'}(s) \tag{193}$$

$$= \frac{1}{p(s')} \sum_{t'} \theta_{t'} \sum_{s\in s'} p(s) x_{t'}(s) \tag{194}$$

$$= \frac{1}{p(s')} \sum_{t'} \theta_{t'} \sum_{s\in s'\cap t'} p(s) \tag{195}$$

$$= \frac{1}{p(s')} \sum_{t'} p(s'\cap t') \theta_{t'} \tag{196}$$

$$= \frac{1}{p(s')} \sum_{\tau} \sum_{t'\in\tau} p(s'\cap t') \theta_{t'}. \tag{197}$$

where $\sum_\tau$ denotes a sum over all augmented sequence orbits τ. Now let $l_1, \ldots, l_K, m_1, \ldots, m_J$ denote the $\mathcal{A}$-positions of s', let $l_1, \ldots, l_K, n_1, \ldots, n_I$ denote the $\mathcal{A}$-positions of τ, and assume $l_1, \ldots, l_K, m_1, \ldots, m_J, n_1, \ldots, n_I$ are distinct. Then for each orbit τ,

$$\sum_{t'\in\tau} p(s'\cap t')\theta_{t'} = \sum_{t'_{l_1}\cdots t'_{l_K}} \sum_{t'_{n_1}\cdots t'_{n_I}} p_{l_1}^{s'_{l_1}} \cdots p_{l_K}^{s'_{l_K}} p_{m_1}^{s'_{m_1}} \cdots p_{m_J}^{s'_{m_J}} p_{n_1}^{t'_{n_1}} \cdots p_{n_I}^{t'_{n_I}} \delta_{s'_{l_1} t'_{l_1}} \cdots \delta_{s'_{l_K} t'_{l_K}} \theta_{l_1\cdots l_K n_1\cdots n_I}^{t'_{l_1}\cdots t'_{l_K} t'_{n_1}\cdots t'_{n_I}} \tag{198}$$

$$= p_{l_1}^{s'_{l_1}} \cdots p_{l_K}^{s'_{l_K}} p_{m_1}^{s'_{m_1}} \cdots p_{m_J}^{s'_{m_J}} \sum_{t'_{l_1} \cdots t'_{l_K}} \delta_{s'_{l_1} t'_{l_1}} \cdots \delta_{s'_{l_K} t'_{l_K}} \sum_{t'_{n_1} \cdots t'_{n_I}} p_{n_1}^{t'_{n_1}} \cdots p_{n_I}^{t'_{n_I}} \theta_{l_1 \cdots l_K n_1 \cdots n_I}^{t'_{l_1} \cdots t'_{l_K} t'_{n_1} \cdots t'_{n_I}}. \quad (199)$$

Noting that

$$\delta_{s'_{l_1} t'_{l_1}} \cdots \delta_{s'_{l_K} t'_{l_K}} = \begin{cases} 1 & \text{if } s'_l = t'_l \text{ for all } l = l_1, \dots, l_K, \\ 0 & \text{otherwise,} \end{cases} \quad (200)$$

and that by Claim 21,

$$\sum_{t'_{n_1} \cdots t'_{n_I}} p_{n_1}^{t'_{n_1}} \cdots p_{n_I}^{t'_{n_I}} \theta_{l_1 \cdots l_K n_1 \cdots n_I}^{t'_{l_1} \cdots t'_{l_K} t'_{n_1} \cdots t'_{n_I}} = \begin{cases} \theta_{l_1 \cdots l_K}^{t'_{l_1} \cdots t'_{l_K}} & \text{if } I = 0, \\ 0 & \text{otherwise,} \end{cases} \quad (201)$$

we see that,

$$\sum_{t' \in \tau} p(s' \cap t') \theta_{t'} = p_{l_1}^{s'_{l_1}} \cdots p_{l_K}^{s'_{l_K}} p_{m_1}^{s'_{m_1}} \cdots p_{m_J}^{s'_{m_J}} \sum_{t'_{l_1} \cdots t'_{l_K}} \theta_{l_1 \cdots l_K}^{t'_{l_1} \cdots t'_{l_K}} \times \begin{cases} 1 \text{ if } t'_l \in \mathcal{A} \Rightarrow s'_l = t'_l, \text{ for all } l = l_1, \dots, l_K, \\ 0 \text{ otherwise.} \end{cases} \quad (202)$$

$$= p(s') \sum_{t' \in \tau} \theta_{t'} \begin{cases} 1 \text{ if } s' \subseteq t', \\ 0 \text{ otherwise.} \end{cases} \quad (203)$$

Consequently,

$$\langle f | s' \rangle_p = \frac{1}{p(s')} \sum_{\tau} p(s') \sum_{t' \in \tau} \theta_{t'} \begin{cases} 1 \text{ if } s' \subseteq t', \\ 0 \text{ otherwise,} \end{cases} \quad (204)$$

$$= \sum_{t'} \theta_{t'} \begin{cases} 1 \text{ if } s' \subseteq t', \\ 0 \text{ otherwise,} \end{cases} \quad (205)$$

$$= \sum_{t' \supseteq s'} \theta_{t'}. \quad (206)$$

This completes the proof for the case where p is positive. The proof for non-positive p follows from the definition and the continuity of projection matrix elements $P_{s't'}^{\infty,p}$ (Eq. 176) with respect to p and the definition $\langle f|s'\rangle_p = \lim_{\epsilon \to 0^+} \langle f|s'\rangle_{p_\epsilon}$. □

Definition 24. *The orbital component of an activity landscape $f = \sum_{s'} \theta_{s'} x_{s'}$ corresponding to an augmented sequence orbit σ is defined to be $f_\sigma(s) = \sum_{s' \in \sigma} \theta_{s'} x_{s'}(s)$.*

Claim 23. *Given an activity landscape f, and augmented sequence orbits σ and τ,*

$$\langle f_\sigma \rangle_p = \begin{cases} \theta_0 & \text{if } o(\sigma) = 0, \\ 0 & \text{otherwise,} \end{cases} \quad (207)$$

and $\langle f_\sigma f_\tau \rangle_p = \delta_{\sigma\tau} \langle f_\sigma^2 \rangle_p$ where

$$\langle f_\sigma^2 \rangle_p = \sum_{s' \in \sigma} p(s') \theta_{s'}^2. \quad (208)$$

Proof. Eq. 207 follows directly from Claim 21:

$$\langle f_\sigma \rangle_p = \sum_{s' \in \sigma} p(s') \theta_{s'} \begin{cases} \theta_0 & \text{if } o(\sigma) = 0, \\ 0 & \text{otherwise,} \end{cases} \quad (209)$$

Let $l_1, \dots, l_K, m_1, \dots, m_J$ denote the $\mathcal{A}$-positions of σ, let $l_1, \dots, l_K, n_1, \dots, n_I$ denote the $\mathcal{A}$-positions of τ, and assume $l_1, \dots, l_K, m_1, \dots, m_J, n_1, \dots, n_I$ are distinct. Then,

$$\langle f_\sigma f_\tau \rangle_p = \sum_u p(u) \left[\sum_{s' \in \sigma} \theta_{s'} x_{s'}(u) \right] \left[\sum_{t' \in \tau} \theta_{t'} x_{t'}(u) \right] \quad (210)$$

$$= \sum_{s' \in \sigma} \sum_{t' \in \tau} \theta_{s'} \theta_{t'} \sum_u p(u) x_{s' \cap t'}(u) \quad (211)$$

$$= \sum_{s' \in \sigma} \sum_{t' \in \tau} p(s' \cap t') \theta_{s'} \theta_{t'} \quad (212)$$

$$= \sum_{s'_{l_1} \cdots s'_{l_K}} \sum_{s'_{m_1} \cdots s'_{m_J}} \sum_{t'_{l_1} \cdots t'_{l_K}} \sum_{t'_{n_1} \cdots t'_{n_I}} p_{l_1}^{s'_{l_1}} \cdots p_{l_K}^{s'_{l_K}} p_{m_1}^{s'_{m_1}} \cdots p_{m_J}^{s'_{m_J}} p_{n_1}^{t'_{n_1}} \cdots p_{n_I}^{t'_{n_I}} \times \quad (213)$$

$$\delta_{s'_{l_1}t'_{l_1}}\cdots\delta_{s'_{l_K}t'_{l_K}}\theta^{s'_{l_1}\cdots s'_{l_K}s'_{m_1}\cdots s'_{m_J}}_{l_1\cdots l_K m_1\cdots m_J}\theta^{t'_{l_1}\cdots t'_{l_K}t'_{n_1}\cdots t'_{n_I}}_{l_1\cdots l_K n_1\cdots n_I} \tag{214}$$

$$= \sum_{s'_{l_1}\cdots s'_{l_K}}\sum_{t'_{l_1}\cdots t'_{l_K}} p^{s'_{l_1}}_{l_1}\cdots p^{s'_{l_K}}_{l_K}\delta_{s'_{l_1}t'_{l_1}}\cdots\delta_{s'_{l_K}t'_{l_K}}\times \tag{215}$$

$$\left[\sum_{s'_{m_1}\cdots s'_{m_J}} p^{s'_{m_1}}_{m_1}\cdots p^{s'_{m_J}}_{m_J}\theta^{s'_{l_1}\cdots s'_{l_K}s'_{m_1}\cdots s'_{m_J}}_{l_1\cdots l_K m_1\cdots m_J}\right]\times\left[\sum_{t'_{n_1}\cdots t'_{n_I}} p^{t'_{n_1}}_{n_1}\cdots p^{t'_{n_I}}_{n_I}\theta^{t'_{l_1}\cdots t'_{l_K}t'_{n_1}\cdots t'_{n_I}}_{l_1\cdots l_K n_1\cdots n_I}\right]. \tag{216}$$

Because

$$\sum_{s'_{m_1}\cdots s'_{m_J}} p^{s'_{m_1}}_{m_1}\cdots p^{s'_{m_J}}_{m_J}\theta^{s'_{l_1}\cdots s'_{l_K}s'_{m_1}\cdots s'_{m_J}}_{l_1\cdots l_K m_1\cdots m_J} = \begin{cases}\theta^{s'_{l_1}\cdots s'_{l_K}}_{s_1\cdots s_K} & \text{if } J=0,\\ 0 & \text{otherwise,}\end{cases} \tag{217}$$

and

$$\sum_{t'_{n_1}\cdots t'_{n_I}} p^{t'_{n_1}}_{n_1}\cdots p^{t'_{n_I}}_{n_I}\theta^{t'_{l_1}\cdots t'_{l_K}t'_{n_1}\cdots t'_{n_I}}_{l_1\cdots l_K n_1\cdots n_I} = \begin{cases}\theta^{t'_{l_1}\cdots t'_{l_K}}_{l_1\cdots l_K} & \text{if } I=0,\\ 0 & \text{otherwise,}\end{cases} \tag{218}$$

we see that the summand in Eq. 215 vanishes unless $J=0$ and $I=0$. This is equivalent to the requirement that $\sigma=\tau$. Therefore,

$$\begin{aligned}\langle f_\sigma f_\tau\rangle_p &= \delta_{\sigma\tau}\sum_{s'_{l_1}\cdots s'_{l_K}}\sum_{t'_{l_1}\cdots t'_{l_K}} p^{s'_{l_1}}_{l_1}\cdots p^{s'_{l_K}}_{l_K}\delta_{s'_{l_1}t'_{l_1}}\cdots\delta_{s'_{l_K}t'_{l_K}}\theta^{s'_{l_1}\cdots s'_{l_K}}_{s_1\cdots s_K}\theta^{t'_{l_1}\cdots t'_{l_K}}_{l_1\cdots l_K} && (219)\\ &= \delta_{\sigma\tau}\sum_{s'\in\sigma}\sum_{t'\in\tau} p(s')\delta_{s't'}\theta_{s'}\theta_{t'} && (220)\\ &= \delta_{\sigma\tau}\sum_{s'\in\sigma} p(s')\theta^2_{s'} && (221)\\ &= \delta_{\sigma\tau}\left\langle f^2_\sigma\right\rangle_p && (222)\end{aligned}$$

where

$$\left\langle f^2_\sigma\right\rangle_p = \sum_{s'\in\sigma} p(s')\theta^2_{s'}. \tag{223}$$

□

Definition 25. *Given $k\in\{0,1,\ldots,L\}$, the k'the order component of an activity landscape $f=\sum_{s'}\theta_{s'}x_{s'}$ is defined to be*

$$f_k(s) = \sum_{s':o(s')=k}\theta_{s'}x_{s'}(s). \tag{224}$$

Claim 24. *Given an activity landscape $f=\sum_{s'}\theta_{s'}x_{s'}$, and parameters $\vec{\theta}$ expressed in the hierarchical gauge,*

$$\text{var}_p[f] = \sum_{k=0}^{L}\text{var}_p[f_k], \quad \textit{where} \quad \text{var}_p[f_k] = \begin{cases}\sum_{s':o(s')=k} p(s')\theta^2_{s'} & \textit{if } \ k\geq 1,\\ 0 & \textit{if } \ k=0.\end{cases} \tag{225}$$

Proof. First we decompose each f_k into a sum of f_σ over augmented sequence orbits σ of order k:

$$f_k = \sum_{\sigma:o(\sigma)=k} f_\sigma. \tag{226}$$

next, by Claim 23,

$$\langle f_k\rangle_p = \sum_{\sigma:o(\sigma)=k}\langle f_\sigma\rangle_p, \tag{227}$$

and

$$\begin{aligned}\left\langle f^2_k\right\rangle_p &= \left\langle\sum_{\sigma:o(\sigma)=k} f_\sigma\sum_{\tau:o(\tau)=k} f_\tau\right\rangle_p && (228)\\ &= \sum_{\sigma:o(\sigma)=k}\sum_{\tau:o(\tau)=k}\langle f_\sigma f_\tau\rangle_p && (229)\end{aligned}$$

$$= \sum_{\sigma:o(\sigma)=k} \sum_{\tau:o(\tau)=k} \delta_{\sigma\tau} \left\langle f_\sigma^2 \right\rangle \tag{230}$$

$$= \sum_{\sigma:o(\sigma)=k} \left\langle f_\sigma^2 \right\rangle \tag{231}$$

$$= \sum_{\sigma:o(\sigma)=k} \sum_{s'\in\sigma} p(s')\theta_{s'}^2 \tag{232}$$

$$= \sum_{s':o(s')=k} p(s')\theta_{s'}^2. \tag{233}$$

Consequently

$$\begin{aligned}
\text{var}_p[f_k] &= \left\langle f_k^2 \right\rangle_p - \left\langle f_k \right\rangle_p^2 & (234)\\
&= \sum_{s':o(s')=k} p(s')\theta_{s'}^2 - \delta_{k0}\theta_0^2 & (235)\\
&= \begin{cases} \sum_{s':o(s')=k} p(s')\theta_{s'}^2 & \text{if } \; k \geq 1, \\ 0 & \text{if } \; k = 0. \end{cases} & (236)
\end{aligned}$$

Finally,

$$\begin{aligned}
\text{var}_p[f] &= \left\langle f^2 \right\rangle_p - \left\langle f \right\rangle_p^2 & (237)\\
&= \left\langle \sum_\sigma f_\sigma \sum_\tau f_\tau \right\rangle_p - \left\langle \sum_\sigma f_\sigma \right\rangle_p^2 & (238)\\
&= \sum_\sigma \sum_\tau \left\langle f_\sigma f_\tau \right\rangle - \sum_\sigma \left\langle f_\sigma \right\rangle_p^2 & (239)\\
&= \sum_\sigma \left[\left\langle f_\sigma^2 \right\rangle_p - \left\langle f_\sigma \right\rangle^2 \right] & (240)\\
&= \sum_{k=0}^{L} \sum_{\sigma:o(\sigma)=k} \left[\left\langle f_\sigma^2 \right\rangle_p - \left\langle f_\sigma \right\rangle^2 \right] & (241)\\
&= \sum_{k=0}^{L} \text{var}_p[f_k]. & (242)
\end{aligned}$$

This completes the proof. □

8 Hierarchical gauges of an empirical landscape for protein GB1

Gauge-fixing formula for the all-order interaction model Using the formula for $P^{\infty,p}$ in Eq. 176 to compute

$$\vec{\theta}_{\text{fixed}} = P^{\infty,p}\vec{\theta} \tag{243}$$

for an all-order interaction model in which only the zero-order, first-order, and second-order parameters are nonzero, one finds that

$$\begin{aligned}
\theta_{0,\text{fixed}} &= \theta_0 + \sum_l \sum_c p_l^c \theta_l^c + \sum_l \sum_{l'>l} \sum_{c,c'} p_l^c p_{l'}^{c'} \theta_{ll'}^{cc'}, & (244)\\
\theta_{l,\text{fixed}}^c &= \sum_{c'} (\delta_{cc'} - p_l^{c'})\theta_l^c + \sum_{l'<l} \sum_{c',c''} (\delta_{cc'} - p_l^{c'}) p_{l'}^{c''} \theta_{ll'}^{cc'} + \sum_{l'>l} \sum_{c',c''} (\delta_{cc'} - p_l^{c'}) p_{l'}^{c''} \theta_{l'l}^{c'c}, & (245)\\
\theta_{ll',\text{fixed}}^{cc'} &= \sum_{c'',c'''} (\delta_{cc''} - p_l^{c''})(\delta_{c'c'''} - p_{l'}^{c'''})\theta_{ll'}^{c''c'''}, & (246)\\
\theta_{l_1 \ldots l_K,\text{fixed}}^{c_1 \ldots c_K} &= 0 \quad \text{for all} \quad K = 3, \ldots, L. & (247)
\end{aligned}$$

Ignoring the formula for parameters of order three or greater, one thus obtains the gauge-fixing formulae for the parameters of the pairwise-interaction model. These are the formulas used for the computations in Fig. 4 and Fig. 5 of the main text. The specific choices for p used in these figures are given below.

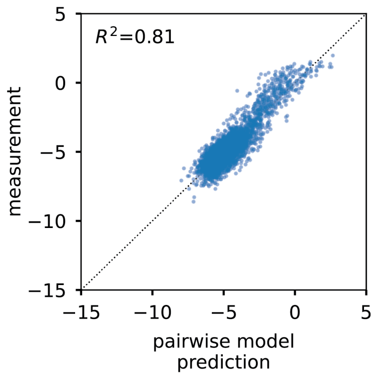

Figure S1: Performance of the pairwise-interaction model for protein GB1. Axes reflect $\log_2$ enrichment ratios. Each dot represents a randomly chosen variant GB1 protein assayed by [2]. For clarity, only 5,000 of the ∼160,000 assayed GB1 variants are shown.

Region-specific distributions. In Fig. 4D, the probability distributions $p(s) = \prod_{l=1}^{4} p_l^{s_l}$ for the four different regions (global, region 1, region 2, region 3) were defined as follows.

- Uniform: For $l \in \{1, 2, 3, 4\}$, $p_l^c = \frac{1}{20}$.
- Region 1: For $l \in \{1, 2, 4\}$, $p_l^c = \frac{1}{20}$; for $l = 3$, $p_l^c = \delta_{c\text{G}}$.
- Region 2: For $l \in \{1, 2\}$, $p_l^c = \frac{1}{20}$; for $l = 3$, $p_l^c = \frac{1}{2}\delta_{c\text{L}} + \frac{1}{2}\delta_{c\text{F}}$; for $l = 4$, $p_l^c = \delta_{c\text{G}}$.
- Region 3: For $l \in \{1, 2\}$, $p_l^c = \frac{1}{20}$; for $l = 3$, $p_l^c = \frac{1}{2}\delta_{c\text{C}} + \frac{1}{2}\delta_{c\text{A}}$; for $l = 4$, $p_l^c = \delta_{c\text{A}}$.

Here $l = 1, 2, 3, 4$ are used to denote protein positions $39, 40, 41, 54$, respectively, and c indexes all $\alpha = 20$ possible amino acids in $\mathcal{A}$.

9 Appendix

Claim 25. *Let V_1 and V_2 be two subspaces of a vector space V such that any vector in V can be uniquely decomposed into the sum of a vector in V_1 and a vector in V_2. Let P_1 be the projection into V_1 along V_2, and P_2 be the projection into V_2 along V_1. Let Λ be a symmetric positive definite matrix acting on V. Then the following three statements are equivalent.*

1. *V_1 and V_2 are Λ-orthogonal, i.e., $\vec{v}_1^\top \Lambda \vec{v}_2 = 0$ for all $\vec{v}_1 \in V_1$ and $\vec{v}_2 \in V_2$.*
2. *For any fixed $\vec{v}_1 \in V_1$, $\text{argmin}_{\vec{v}_2 \in V_2} (\vec{v}_1 + \vec{v}_2)^\top \Lambda (\vec{v}_1 + \vec{v}_2) = \vec{0}$.*
3. *$\Lambda = P_1^\top \Lambda P_1 + P_2^\top \Lambda P_2$.*

Proof. We prove equivalence of the three statements (denoted 1, 2, and 3) as follows.

- $1 \Rightarrow 2$: Assume that 1 is true. Then for all $\vec{v}_1 \in V_1$ and $\vec{v}_2 \in V_2$, $(\vec{v}_1 + \vec{v}_2)^\top \Lambda (\vec{v}_1 + \vec{v}_2) = \vec{v}_1^\top \Lambda \vec{v}_1 + \vec{v}_2^\top \Lambda \vec{v}_2 \geq \vec{v}_1^\top \Lambda \vec{v}_1$. Because equality obtains only when $\vec{v}_2 = \vec{0}$, $\vec{0}$ is the unique $\vec{v}_2 \in V$ that minimizes $(\vec{v}_1 + \vec{v}_2)^\top \Lambda (\vec{v}_1 + \vec{v}_2)$. This proves 2, thereby establishing that $1 \Rightarrow 2$.
- $2 \Rightarrow 1$: Assume that 1 is not true, i.e., there exists $\vec{v}_1 \in V_1$ and $\vec{v}_2 \in V_2$ such that $\vec{v}_1^\top \Lambda \vec{v}_2 \neq 0$. Then $\frac{d}{d\epsilon}(\vec{v}_1 + \epsilon \vec{v}_2)^\top \Lambda (\vec{v}_1 + \epsilon \vec{v}_2) = 2\vec{v}_1^\top \Lambda \vec{v}_2 + 2\epsilon \vec{v}_2^\top \Lambda \vec{v}_2$ is nonzero at $\epsilon = 0$. This contradicts 2, thereby establishing that $\neg 1 \Rightarrow \neg 2$ and hence $2 \Rightarrow 1$.
- $1 \Rightarrow 3$: Assume that 1 is true, and choose any $\vec{v}, \vec{w} \in V$. Then $\vec{v}^\top \Lambda \vec{w} = (P_1\vec{v} + P_2\vec{v})^\top \Lambda (P_1\vec{w} + P_2\vec{w}) = (P_1\vec{v})^\top \Lambda (P_1\vec{w}) + (P_2\vec{v})^\top \Lambda (P_2\vec{w}) = \vec{v}^\top \left[P_1^\top \Lambda P_1 + P_2^\top \Lambda P_2 \right] \vec{w}$. This proves 3, thereby establishing that $1 \Rightarrow 3$.
- $3 \Rightarrow 1$: Assume that 3 is true. Then given any $\vec{v}_1 \in V_1$ and $\vec{v}_2 \in V_2$, $\vec{v}_1{}^\top \Lambda \vec{v}_2 = (P_1\vec{v}_1)^\top \Lambda (P_1\vec{v}_2) + (P_2\vec{v}_1)^\top \Lambda (P_2\vec{v}_2) = 0$ since $P_1\vec{v}_2 = P_2\vec{v}_1 = \vec{0}$. This proves 1, thereby establishing that $3 \Rightarrow 1$.

□

References

[1] Anna Posfai, David M. McCandlish, and Justin B. Kinney. Symmetry, gauge freedoms, and the interpretability of sequence-function relationships. *bioRxiv*, 2024.

[2] Nicholas C Wu, Lei Dai, C Anders Olson, James O Lloyd-Smith, and Ren Sun. Adaptation in protein fitness landscapes is facilitated by indirect paths. *eLife*, 5:1965, 07 2016.
